# Comparative multi-omic analyses of cardiac mitochondrial stress in three mouse models of frataxin deficiency

**DOI:** 10.1242/dmm.050114

**Published:** 2023-10-09

**Authors:** Nicole M. Sayles, Jill S. Napierala, Josef Anrather, Nadège Diedhiou, Jixue Li, Marek Napierala, Hélène Puccio, Giovanni Manfredi

**Affiliations:** ^1^ Feil Family Brain and Mind Research Institute, Weill Cornell Medicine, 407 East 61st Street, New York, NY 10065, USA; ^2^ Neuroscience Graduate Program, Will Cornell Graduate School of Medical Sciences, 1300 York Ave, New York, NY 10065, USA; ^3^ Department of Biochemistry and Molecular Genetics, University of Alabama at Birmingham, Birmingham, AL 35294, USA; ^4^ Institut de Génétique et de Biologie Moléculaire et Cellulaire (IGBMC), CNRS/Université de Strasbourg UMR7104, Inserm U1258, B. P. 163, 67404 Illkirch, France; ^5^ Department of Neurology, Peter O'Donnell Jr. Brain Institute, University of Texas Southwestern Medical Center, Dallas, TX 75390, USA

**Keywords:** Friedreich ataxia, Cardiomyopathy, Integrated stress response, Mitochondria, Frataxin, Mouse model

## Abstract

Cardiomyopathy is often fatal in Friedreich ataxia (FA). However, FA hearts maintain adequate function until advanced disease stages, suggesting initial adaptation to the loss of frataxin (FXN). Conditional cardiac knockout mouse models of FXN show transcriptional and metabolic profiles of the mitochondrial integrated stress response (ISR^mt^), which could play an adaptive role. However, the ISR^mt^ has not been investigated in models with disease-relevant, partial decrease in FXN. We characterized the heart transcriptomes and metabolomes of three mouse models with varying degrees of FXN depletion: YG8-800, KIKO-700 and FXN^G127V^. Few metabolites were changed in YG8-800 mice, which did not provide a signature of cardiomyopathy or ISR^mt^; several metabolites were altered in FXN^G127V^ and KIKO-700 hearts. Transcriptional changes were found in all models, but differentially expressed genes consistent with cardiomyopathy and ISR^mt^ were only identified in FXN^G127V^ hearts. However, these changes were surprisingly mild even at advanced age (18 months), despite a severe decrease in FXN levels to 1% of those of wild type. These findings indicate that the mouse heart has low reliance on FXN, highlighting the difficulty in modeling genetically relevant FA cardiomyopathy.

## Teaser

**Summary:** The mitochondrial integrated stress response in the heart of a mouse model of Friedreich ataxia is surprisingly mild, despite a severe decrease in frataxin levels below 1% of normal.

## INTRODUCTION

Heart disease in Friedreich ataxia (FA), a genetic neurodegenerative disease caused by decreased levels of frataxin (FXN) protein, is the leading cause of mortality (Payne, 2022; Tsou et al., 2011). FXN deficiency is due to a trinucleotide repeat expansion (GAA) within the first intron of the *FXN* gene (Campuzano et al., 1996). In 4% of cases, FA is associated with compound heterozygosity, with one expanded allele and one allele harboring a point mutation or a deletion. Interestingly, a G130V substitution has been associated with forms of FA with milder clinical presentation and slower progression (Bidichandani et al., 1997; Galea et al., 2016). FXN is a mitochondrial protein regulating the biogenesis of iron-sulfur (Fe-S) clusters, important co-factors of enzymes involved in many essential biological functions, including the mitochondrial respiratory chain, heme and lipoid acid biosynthesis, iron metabolism and DNA repair (Maio et al., 2020). FXN deficiency leads to impaired Fe-S biogenesis, leading to mitochondrial dysfunction, iron metabolism dysregulation, increased oxidative stress and, eventually, cellular dysfunction and death (Chandran et al., 2017; Puccio et al., 2001).

Approximately 60% of FA patients develop a left ventricular (LV) hypertrophic cardiomyopathy (HCM), often concentric in nature and non-obstructive. FA patients with cardiomyopathy display impaired myocardial perfusion reserve index associated with microvascular alterations and significant cardiac fibrosis (Raman et al., 2011). As the disease progresses, LV wall thinning leads to dilated cardiomyopathy, and arrhythmias are also common and can contribute to mortality (Lees et al., 2022). The hypertrophy is associated with troponin leak, suggesting ongoing injury and damage. The energy production is abnormal, with 40% decrease in cardiac creatine phosphate/adenosine triphosphate (ATP) levels in FA patients compared to those in controls (Lodi et al., 2001). At the histological level, the FA heart presents with fiber hypertrophy associated with mitochondrial proliferation, lipid droplet accumulation, iron deposit, fiber necrosis, fibrosis and inflammatory infiltration (Koeppen, 2011).

Typically, death from cardiomyopathy in FA occurs in the third or fourth decade of life. Surprisingly, the FA heart often maintains adequate systolic function until shortly before death, even though the underlying causes of tissue degeneration – oxidative phosphorylation (OXPHOS) dysfunction, impaired iron homeostasis and oxidative stress – are likely to be present early on, possibly even during development (Palau, 2001). This suggests that the FA heart can adapt, at least initially, to the OXPHOS defects caused by the loss of FXN. This adaptation likely involves metabolic rewiring to allow the utilization of alternative energy sources, which do not depend on OXPHOS.

The normal heart relies heavily on fatty acids as the main energy source (Lopaschuk et al., 2010). However, the enzymatic steps required to generate ATP from fatty acids are defective in FA, owing to Fe-S cluster deficiency (Rotig et al., 1997). Therefore, the FA heart shifts its metabolism towards aerobic glycolysis (Payne and Wagner, 2012). This process is bioenergetically less efficient than mitochondrial OXPHOS, but it can be tolerated if enough glucose is available for glycolysis. Furthermore, there is accumulation of unutilized lipids in cardiomyocytes as well as a build-up of oxidative stress, presumably initiated in mitochondria (Chandran et al., 2017). This complex metabolic adaptation occurring in the FA heart recapitulates the main metabolic features of a process that has been well characterized in mitochondrial diseases, defined as the mitochondrial integrated stress response (ISR^mt^) (Dogan et al., 2014; Forsstrom et al., 2019; Kaspar et al., 2021; Khan et al., 2017; Kuhl et al., 2017; Nikkanen et al., 2016; Sayles et al., 2022).

The ISR^mt^ can occur as a result of protein misfolding in mitochondria (Narayana Rao et al., 2022). It induces activating transcription factor (ATF)4 and ATF5, which downregulate the transcription of many genes, while upregulating the expression of genes involved in proteostasis. The ISR^mt^ also involves a profound rewiring of cellular metabolism, including upregulation of serine one-carbon (1C) metabolism for glutathione production (Mehrmohamadi et al., 2014; Nikkanen et al., 2016). Furthermore, the ISR^mt^ stimulates the production of myokines, such as GDF15 and FGF21, which are secreted into the blood stream and signal to the liver and the adipose tissue to activate gluconeogenesis and fatty acid mobilization (Boenzi and Diodato, 2018).

There is evidence that ISR^mt^ activation occurs in the hearts of mouse models of *Fxn* genetic ablation. In a heart-specific mouse model with conditional knockout (cKO) of *Fxn*, there was elevation of ATF4 accompanied by suppression of protein synthesis, elevation of chaperones and proteases, and upregulation of serine-1C metabolism (Huang et al., 2013). Furthermore, serine-1C metabolism rewiring and FGF21 upregulation were described in the heart of a mouse model with inducible silencing of *Fxn* (Vasquez-Trincado et al., 2021). However, these studies examined mouse models that do not fully reflect the physiology of FA in humans, because the complete genetic ablation of *Fxn* in the heart or the acute postnatal silencing of *Fxn* differ from the situation in the human heart, in which very low levels of FXN are present since embryonic development. Therefore, to assess the effects of heart FXN deficiency in more physiological model systems, we investigated three independent mouse models with varying degrees of constitutive low levels of FXN using an integrated multi-omic approach. We studied a mouse harboring a human GAA expanded (800 GAA repeats) *Fxn* transgene in a mouse *Fxn* knockout (KO) background (YG8-800), a mouse containing an intron 1 GAA expansion (700 repeats) in the endogenous *Fxn* gene with genetic ablation of the other allele (KIKO-700), and a mouse with a homozygous knock-in (KI) *Fxn* missense mutation resulting in a G127V amino acid substitution, the mouse equivalent of the human G130V variant (FXN^G127V^) (Bidichandani et al., 1997; Galea et al., 2016). This approach allows the comparison of different models of FXN depletion with distinct genetic underpinnings.

## RESULTS

### Gene expression profiles indicate mitochondrial and cardiac stress in the YG8-800 mouse

To investigate whether heart ISR^mt^ and related metabolic rewiring occur in a mouse model of partial FXN deficiency, we performed unbiased metabolomic and transcriptomic analyses in the heart of a humanized FA model, the Tg(FXN)YG8Pook/800J (YG8-800) mouse from The Jackson Laboratory. YG8-800 transgenic mice are homozygous for a *Fxn* constitutive null allele (exon 2 deletion) and hemizygous for a human *FXN* transgene, which contains >800 GAA repeats in intron 1. Recently, the heart of this mouse model was characterized, and, by enzyme-linked immunosorbent assay (ELISA), the levels of cardiac human FXN were estimated to be ∼5% of those of a control mouse expressing human *FXN* with only nine GAA repeats (Gerard et al., 2023). Cardiac involvement included increased heart-to-body weight ratio and moderately reduced ejection fraction at 6 months of age. At this time, the cardiac phenotype was defined as being at an early stage of progression. Thus, we opted to investigate the metabolomic and transcriptomic profiles of hearts from YG8-800 mice and littermate controls [wild type (WT)], expressing normal levels of mouse FXN and no human FXN, at 18 months of age, expecting that cardiac alterations would be more severe in the aging mouse. By ELISA, we found that the YG8-800 mice expressed lower levels of FXN (21±4 ng/mg protein) compared to endogenous mouse levels in the WT littermate controls (208±16 ng/mg protein) (Fig. S1A). Because no differences were reported between males and females (Gerard et al., 2023), we utilized only males for these studies.

**Fig. 1.**
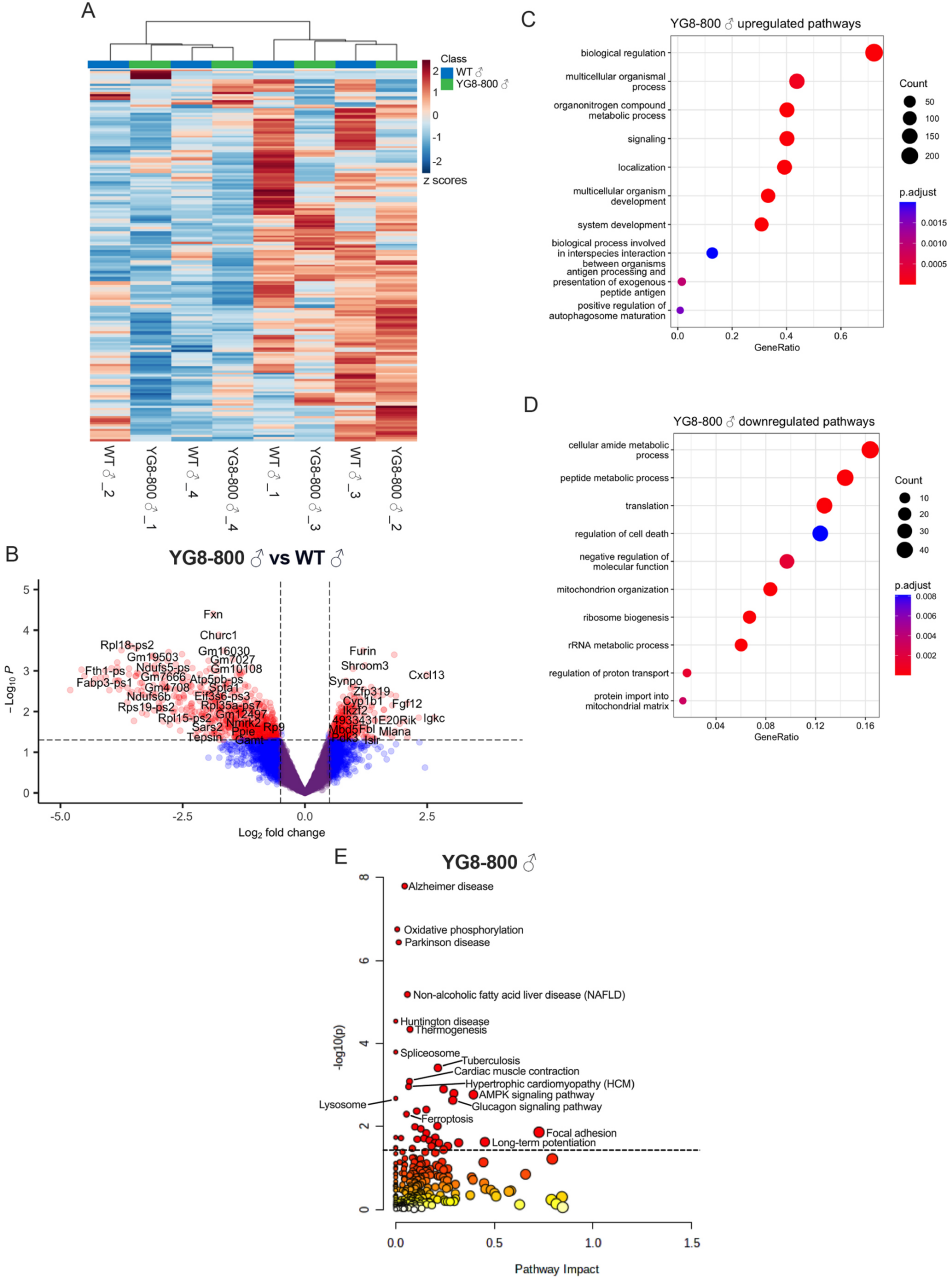
**Enrichment of pathways associated with mitochondrial and cardiac stress in YG8-800 mice.** (A) Heatmap with hierarchical clustering of all measured metabolites from wild-type (WT) and YG8-800 hearts (*n*=4 18-month-old mice). Red indicates increased abundance and blue indicates reduced abundance. (B) Volcano plot of gene expression (*n*=4 18-month-old mice). Genes in red indicate *P*>0.05, |FC|>0.5. (C,D) Top ten results of unbiased Gene Ontology (GO) analyses of significantly upregulated (C) or downregulated (D) genes. Gene ratio is calculated by dividing the number of genes that belong to a given gene set by the total number of genes in the gene set. Count is indicated by size and adjusted *P*-value (p.adjust) is indicated by color. (E) Joint-pathway analysis of integrated metabolomics and transcriptomics data from YG8-800 males. Pathway impact is a combination of the centrality and pathway enrichment results, calculated as the sum of the importance measures of each of the matched metabolites and then divided by the sum of the importance measures of all metabolites in each pathway. For each data point, the size of the circle is correlated with pathway impact and deeper color indicates higher significance. Kyoto Encyclopedia of Genes and Genomes (KEGG) IDs of some significantly enriched pathways [–Log10(*P*)>1.3] were added to the joint-pathway analysis in MetaboAnalyst.

Hierarchical cluster analysis of metabolomic data did not show clustering of samples by genotype (Fig. 1A), indicating there were very few metabolic differences between YG8-800 and WT hearts, with only six metabolites (4-pyridoxic acid, NADH, kynurenic acid, glutathione, deoxyribose 5-phosphate and guanosine triphosphate) reaching a significance threshold (*P*<0.05). Unbiased metabolic Kyoto Encyclopedia of Genes and Genomes (KEGG) pathway analysis by MetaboAnalyst of the six significantly altered metabolites suggested alterations in vitamin B6 metabolism (Fig. S1B, Table S1). Transcriptomic analysis of YG8-800 hearts showed several differentially expressed genes (DEGs; with a threshold set at *P*<0.05) (1052 DEGs), the majority of which were downregulated (Fig. 1B). Specifically, there were 669 downregulated {Log_2_[fold change (FC)]<0} and 383 upregulated [Log_2_(FC)>0] DEGs. Next, we performed unbiased Gene Ontology (GO) analyses of all DEGs [i.e. all genes with *P*<0.05 and without cutoff for Log_2_(FC)], which revealed several significantly altered pathways. The top ten most significant upregulated pathways were broad categories that did not suggest cardiac stress (Fig. 1C). However, among the top ten most significant downregulated pathways, there were several enrichments of interest, including protein translation, ribosome biogenesis, mitochondrion organization and protein import into mitochondrial matrix (Fig. 1D).

Next, we looked at the integrated pathways with MetaboAnalyst joint-pathway analysis of the transcriptomic and metabolomic data. An initial analysis including ribosomal genes resulted in overrepresentation of the ribosome and RNA transport pathways (Fig. S1C, Table S2), which hindered the visualization of other enriched pathways. Therefore, we performed another integrated analysis, excluding ribosomal subunit genes that modified the significance of the remaining pathways, which allowed for better visualization of several pathways related to HCM, ferroptosis and OXPHOS, in addition to pathways associated with neurodegeneration (Fig. 1E; Table S2). Overall, these results suggest cardiac stress without evidence of ISR^mt^.

### KIKO-700 mice display metabolic and transcriptional alterations associated with cardiac stress and ISR^mt^

**Fig. 2.**
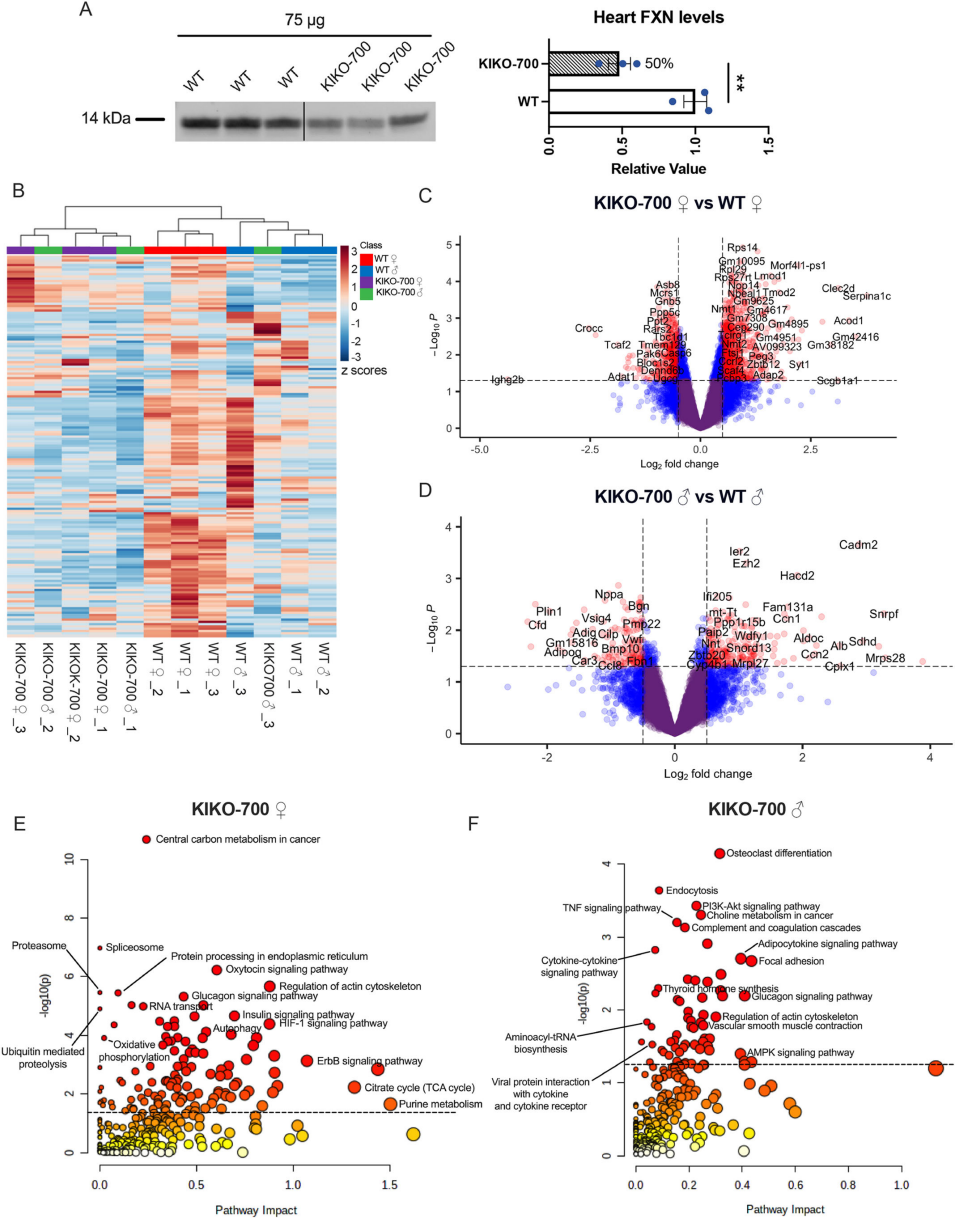
**Enrichment of pathways associated with mitochondrial integrated stress response (ISR^mt^) and cardiac stress in KIKO-700 mice.** (A) Western blot analysis of FXN expression in WT and KIKO-700 male hearts, quantified in the right panel (*n*=3) (statistical analysis was performed with unpaired two-tailed Student's *t*-test, **P<0.009; data are mean±s.e.m.). (B) Heatmap with hierarchical clustering of all measured metabolites from WT and KIKO-700 hearts (*n*=3/sex/genotype 18-month-old mice). (C,D) Volcano plot of gene expression in KIKO-700 females (C) and males (D) (*n*=3/sex/genotype 18-month-old mice). Genes in red indicate *P*>0.05, |FC|>0.5. (E,F) Joint-pathway analysis of integrated metabolomic and transcriptomic data from KIKO-700 females (E) and males (F).

Next, we analyzed another model of partial FXN deficiency, a knock-in, knockout (KIKO) mouse model. In this model, one allele of mouse *Fxn* is deleted, while the other allele harbors a GAA expansion in intron 1. In a well-established FA KIKO mouse model harboring >230 GAA repeats (KIKO-230), ∼30% residual FXN protein is detected in the heart (Miranda et al., 2002). This level of residual FXN was shown to be sufficient to prevent iron accumulation and severe fibrosis in the heart (Miranda et al., 2002). Herein, we investigated the cardiac effects of a newly generated KIKO mouse with a longer GAA expansion, harboring 700 repeats (KIKO-700). Longer expansions in FA patients are associated with earlier disease onset, increased disease severity and LV hypertrophy (Isnard et al., 1997; Koeppen, 2011). Surprisingly, KIKO-700 hearts at 18 months of age had only 50% reduction in FXN protein, as estimated by western blotting normalized by total protein (Fig. 2A; Fig. S2A). Therefore, despite the longer GAA expansion in the KIKO-700 mice, the levels of residual heart FXN are higher than those reported for the KIKO-230 model.

Metabolomic and transcriptomic analyses were performed on male and female KIKO-700 and WT hearts. Hierarchical clustering of detected metabolites revealed partial clustering by genotype (Fig. 2B). To note, most differential metabolites in KIKO-700 hearts (*P*<0.05) were decreased in abundance. Unbiased KEGG metabolic pathway analysis revealed several enriched pathways. In female KIKO-700 hearts, glutathione metabolism, purine metabolism, citric acid cycle (tricarboxylic acid cycle) and several amino acid-related metabolic pathways were significantly enriched (Fig. S3A, Table S1). Although male KIKO-700 hearts had fewer differential metabolites than female KIKO-700 hearts (15 versus 58 metabolites respectively), some of the same pathways were enriched, including purine metabolism, arginine biosynthesis and pentose phosphate (Fig. S3B, Table S1). Of these pathways, glutathione metabolism and nucleotide biosynthesis have been associated with ISR^mt^ and found to be dysregulated in various mouse models of mitochondrial dysfunction and HCM (Dogan et al., 2014; Forsstrom et al., 2019; Kaspar et al., 2021; Khan et al., 2017; Kuhl et al., 2017; Nikkanen et al., 2016; Sayles et al., 2022). Furthermore, several DEGs were identified in KIKO-700 hearts, with a higher number of DEGs in females (1804 DEGs in females and 505 DEGs in males) (Fig. 2C,D). We performed GO analyses of significantly (*P*<0.05) upregulated (1080 DEGs in females and 278 in males) and downregulated (784 DEGs in females and 227 in males) genes and found enriched pathways relevant to cardiac stress, including regulation of innate immune response and regulation of cytokine production (Fig. S3C-F).

Next, we performed integrated pathway analysis of female metabolomes and transcriptomes and, like in YG8-800 male mice, found a highly significant enrichment of the ribosome pathway (Fig. S3G, Table S2). After removal of ribosomal genes from the analysis, we could better detect the enrichment of proteotoxic stress pathways (autophagy, proteasome, ubiquitin mediated proteolysis), OXPHOS and regulation of actin cytoskeleton (Fig. 2E, Table S2). These pathways, as well as PI3K-AKT signaling and vascular smooth muscle contraction, were also enriched in male KIKO-700 hearts (Fig. 2F; Table S2). Collectively, these results suggest that ∼50% reduction in FXN in the KIKO-700 heart is associated with cardiac stress, and metabolic and transcriptional changes consistent with ISR^mt^.

### Severe FXN deficiency in FXN^G127V^ hearts is associated with transcriptional and metabolic alterations suggestive of cardiomyopathy and ISR^mt^

**Fig. 3.**
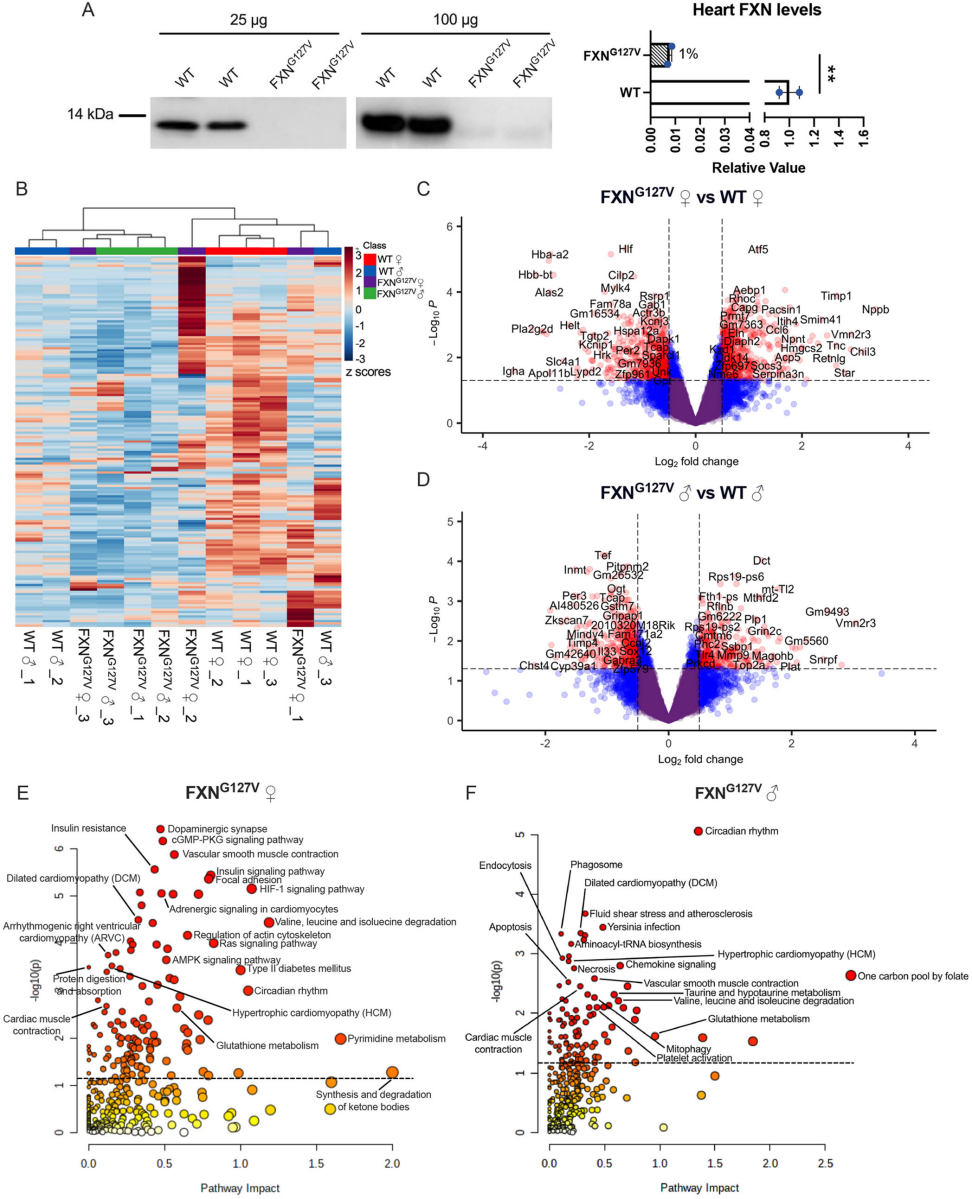
**Enrichment of pathways associated with ISR^mt^ and cardiac stress in FXN^G127V^ mice.** (A) Western blot analyses of FXN expression in WT and FXN^G127V^ male total heart lysates, loading either 25 µg or 100 µg, with the 100 µg blot quantified to the right (*n*=2) (statistical analysis was performed with unpaired two-tailed Student's *t*-test, **P<0.007; data are mean±s.e.m.). (B) Heatmap with hierarchical clustering of all measured metabolites from WT and FXN^G127V^ hearts (*n*=3/sex/genotype 18-month-old mice). (C,D) Volcano plot of gene expression in FXN^G127V^ females (C) and males (D) (*n*=3/sex/genotype 18-month-old mice). Genes in red indicate *P*>0.05, |FC|>0.5. (E,F) Joint-pathway analysis of integrated metabolomic and transcriptomic data from FXN^G127V^ females (E) and males (F).

The third mouse model of FA we analyzed was a homozygous FXN^G127V^ KI, which harbors a missense mutation in exon 4 of both *Fxn* alleles, resulting in a G127V amino acid change (Fil et al., 2020). This variant is equivalent to the human pathogenic G130V found in a small subset of FA patients (Bidichandani et al., 1997; Galea et al., 2016). Mouse embryonic fibroblasts isolated from FXN^G127V^ mice showed severe reduction in FXN levels (5% residual), decreased mitochondrial length and increased mitochondrial DNA (mtDNA) damage (Fil et al., 2020). The central nervous system and the heart of this mouse model have extremely low levels of FXN protein (Fil et al., 2023). FXN^G127V^ mice manifest neurobehavioral deficits and reduced body weight, but whether the heart displays ISR^mt^ has not been investigated. Therefore, we studied the heart of this mouse as a model of severe FXN depletion. First, we confirmed severe FXN reduction by western blot analysis of whole-heart lysates. Using 25 µg of total protein, FXN was undetectable in FXN^G127V^ hearts (Fig. 3A). However, by loading 100 µg of heart lysates, we were able to detect ∼1% residual FXN (Fig. 3A; Fig. S2B).

We performed metabolomic and transcriptomic analyses of male and female hearts from FXN^G127V^ and WT mice at 18 months of age. Hierarchical clustering analysis of FXN^G127V^ and WT heart metabolites showed clustering by genotype (Fig. 3B). In both male and female FXN^G127V^ hearts, all differential metabolites were decreased in abundance. Unbiased metabolic KEGG pathway analysis of the female FXN^G127V^ metabolome found enrichment of metabolites involved in purine, glutathione and amino acid (valine, leucine, isoleucine, arginine) metabolism (Fig. S4A, Table S1). In male FXN^G127V^ hearts, enriched metabolic pathways included glutathione metabolism, pantothenate and CoA biosynthesis, pyrimidine metabolism, and D-glutamine and D-glutamate metabolism (Fig. S4B, Table S1). Transcriptomics revealed many DEGs in both female (1678 DEGs) and male (1089 DEGs) FXN^G127V^ hearts, with overrepresentation in females (Fig. 3C,D). GO analyses of significantly upregulated (856 in females and 514 in males) or downregulated (822 in females and 575 in males) genes showed enriched pathways known to be associated with the ISR^mt^ and cardiac stress, including, for example, response to stress in female FXN^G127V^ hearts (Fig. S4C,D), and immune system and tetrahydrofolate metabolic processes in male FXN^G127V^ hearts (Fig. S4E,F).

Integrated pathway analysis of significantly altered (*P*<0.05) metabolites and transcripts in female FXN^G127V^ hearts revealed significant enrichment of the ribosome pathway (Fig. S4G, Table S2). After removing ribosomal genes from the analysis, several pathways associated with cardiac stress could be visualized, such as dilated cardiomyopathy, HCM, vascular smooth muscle contraction, cardiac muscle contraction and regulation of actin cytoskeleton (Fig. 3E; Table S2). There were also several enriched pathways associated with ISR^mt^, including the 1C pool by folate, glutathione metabolism, mitophagy, amino acid metabolism, pyrimidine metabolism and chemokine signaling. Several of these pathways were also enriched in integrated pathway analysis of FXN^G127V^ male hearts, including HCM, the 1C pool by folate and glutathione metabolism (Fig. 3F; Table S2). Together, these results suggest that the severe reduction in FXN expression in the FXN^G127V^ heart leads to metabolic and transcriptional alterations associated with HCM and ISR^mt^.

### Targeted transcriptomic analyses reveal markers of HCM only in FXN^G127V^ hearts

**Fig. 4.**
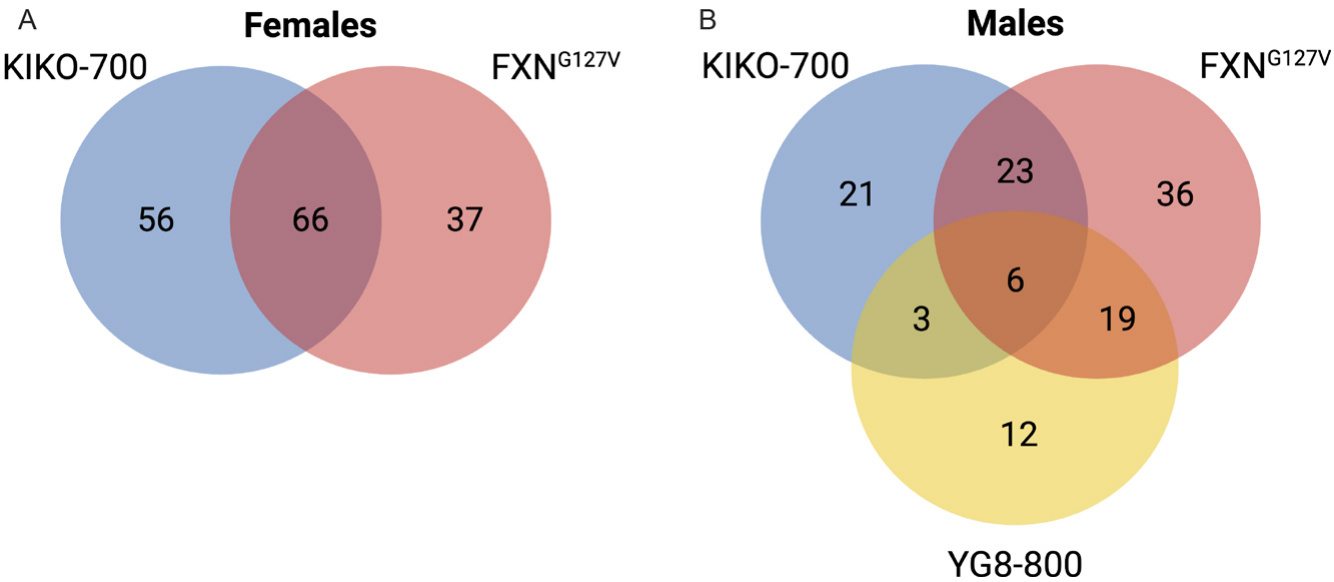
**Distinct pathway enrichment in three models of FXN deficiency.** (A) Venn diagram of joint-pathway analysis results in KIKO-700 and FXN^G127V^ female hearts. (B) Venn diagram of joint-pathway analysis results in KIKO-700, FXN^G127V^ and YG8-800 male hearts.

Based on the unbiased metabolomic and transcriptomic data, the three mouse models of FXN deficiency all shared features of cardiac stress, while pathways consistent with ISR^mt^ were only identified in KIKO-700 and FXN^G127V^ hearts. Next, we compared the enriched integrated pathways in the three models of FXN deficiency in a genotype- and sex-specific manner (except for YG8-800, in which only male mice were analyzed). Most of the pathways were in common between KIKO-700 (54% of total pathways) and FXN^G127V^ (64% of total pathways) female hearts (Fig. 4A; Table S3). Of note, potentially cardiomyopathy-related KEGG pathways included regulation of actin cytoskeleton, HCM, PI3K-AKT signaling and stress signaling pathways, such as FOXO, AMPK and HIF-1 signaling. In males, only six pathways were found to be in common to all three models (apelin signaling, insulin signaling, focal adhesion, Huntington disease, oxytocin signaling and thermogenesis), none of which is directly related to cardiomyopathy or ISR^mt^ (Fig. 4B; Table S3).

To further delve into the cardiac involvement in these mouse models and their relationship to human cardiomyopathies, we performed a targeted analysis of the genes included in the KEGG ‘hypertrophic cardiomyopathy (HCM)’ pathway (pathway mmu05410). This pathway encompasses a total of 91 genes, 70 of which were detected by RNA sequencing (RNAseq) in most groups (except YG8-800 mice, with only 59 genes detected). Percentages of HCM pathway genes that were significantly different (*P*<0.05) relative to those in WT sex-matched hearts were ∼20% in FXN^G127V^ female hearts, 14% in FXN^G127V^ male hearts, 12% in KIKO-700 female hearts, 5% in KIKO-700 male hearts and 14% in YG8-800 male hearts (Fig. S5). Surprisingly, there was little overlap among groups, and none of these DEGs were common to all groups. Overall, these results suggest a sexual dimorphism in the expression of HCM markers in FXN^G127V^ mice; KIKO-700 and YG8-800 mice did not display alterations in these sets of genes. Because the hypertrophic response in FA heart is proposed to result, at least in part, from mitochondrial proliferation (Payne, 2022), we looked at the expression of mitochondrial OXPHOS genes. Overall, we did not observe upregulation of these genes in any of the mouse models investigated (Fig. S6A-E), suggesting that, in these mice, the mitochondrial biogenesis program is not strongly upregulated.

The loss of FXN leads to iron dysregulation in the heart, including increased iron uptake, iron accumulation in mitochondria and ferroptosis (Martelli and Puccio, 2014). To understand the effects of various degrees of FXN depletion on heart iron metabolism, which could underlie cardiomyopathy, we performed targeted gene expression analyses of genes related to iron, including those involved in transferrin-dependent and -independent iron uptake, iron storage and export, and mitochondrial iron import. Only in FXN^G127V^ female hearts did we observe an increase in transferrin receptor (*Tfrc*), ferritin (*Fth1*) and ferroxidase (*Cp*) gene expression relative to that in WT female hearts (Fig. S7A). Furthermore, among genes involved in Fe-S cluster biogenesis and heme metabolism, we found decreased expression of *Nfs1*, *Nubpl* and *Fech* (Fig. S7B,C) only in FXN^G127V^ female hearts. In males, FXN^G127V^ and KIKO-700 hearts had increased expression of aminolevulinic acid synthase 1 (*Alas1*), the heme biosynthesis rate-limiting mitochondrial enzyme, while YG8-800 hearts had increased expression of heme oxygenase-1 (*Hmox1*), which catalyzes the first step in heme degradation (Fig. S7C). Overall, these data indicate that only a few genes involved in iron metabolism are affected in a genotype- and sex-dependent manner, and that these changes are more prominent in FXN^G127V^ female hearts.

**Fig. 5.**
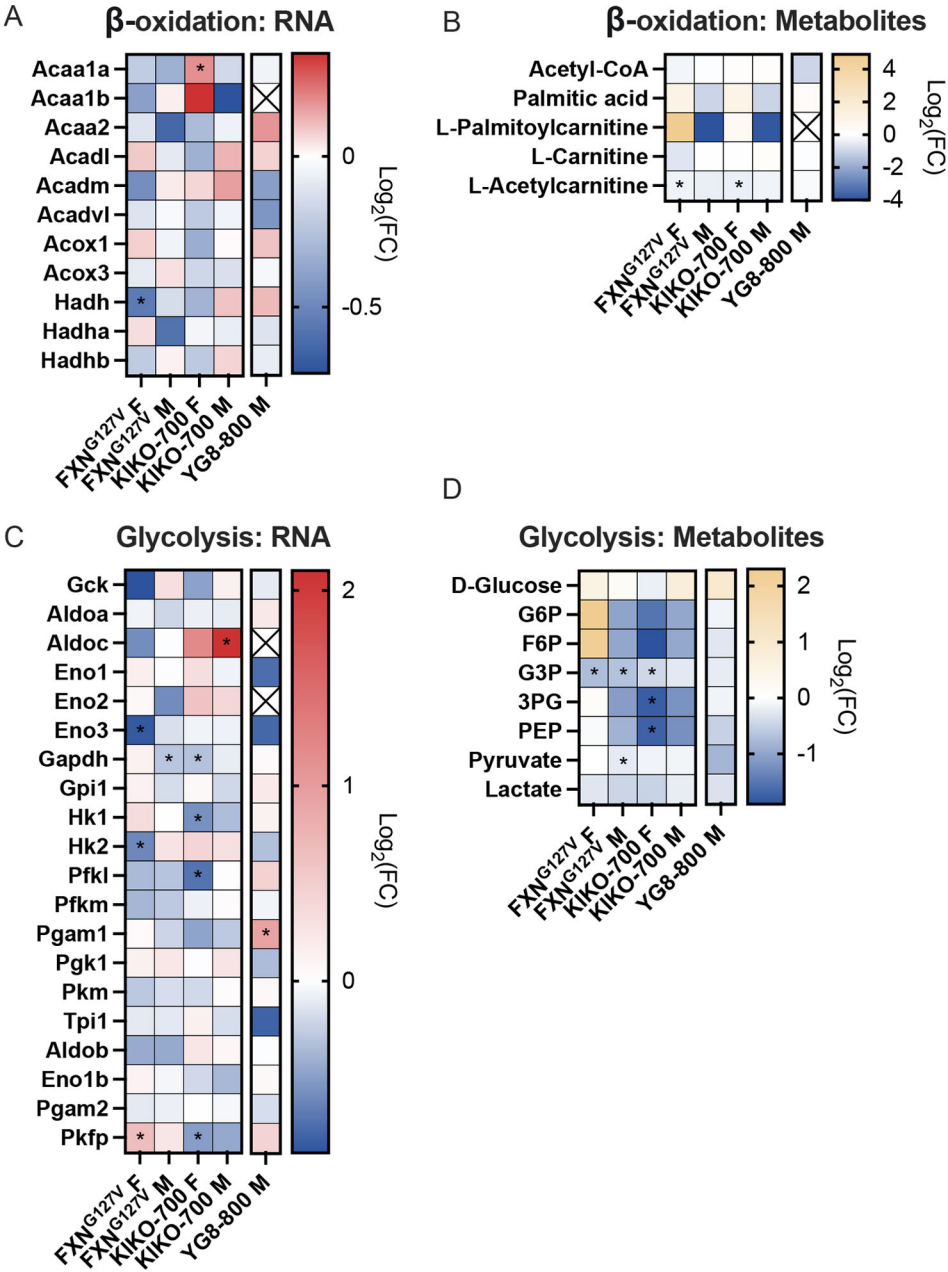
**Normal expression of β-oxidation and glycolysis genes and metabolites in all three FXN-deficiency models.** (A,C) Heatmaps showing the expression of genes related to β-oxidation (KEGG module M00087; A) and glycolysis (KEGG module M00001; C), in which red indicates increased gene expression and blue indicates decreased gene expression. ‘X’ indicates that a gene was not detected by RNAseq. F, female; M, male. (B,D) Heatmaps showing the abundance of metabolites related to β-oxidation (B) and glycolysis (D), in which yellow indicates increased abundance and blue indicates decreased abundance. G6P, glucose-6 phosphate; F6P, fructose-6 phosphate; G3P, glycerol-3-phosphate; 3PG, 3-phosphoglyceric acid; PEP, phosphoenolpyruvic acid. In all heatmaps, expression is represented as Log_2_[fold change (FC)]. *n*=3/sex/genotype 18-month-old FXN^G127V^ and KIKO-800 and *n*=4 18-month-old YG8-800 mice. Transcriptomics statistical analyses were performed with moderated *t*-test. Metabolomics statistical analyses were performed with unpaired two-tailed Student’s *t*-test, *P<0.05.

The adult heart relies primarily on β-oxidation of fatty acids to provide substrates for mitochondrial OXPHOS. Under stress, the heart alters metabolic fuel sources to support contraction, shifting its energy metabolism towards glycolysis (Lopaschuk and Jaswal, 2010; Lopaschuk et al., 2010). The integrated pathway analyses performed on significantly altered metabolites and transcripts of the heart of the three mouse models of FXN deficiency suggested the involvement of several pathways related to energy metabolism (Fig. 1E, Fig. 2E,F and Fig. 3E,F). Therefore, to better define the potential link between cardiomyopathy and energy metabolism, we performed a targeted analysis of the heart transcriptome and metabolome in the three models. We evaluated the expression of genes of the KEGG ‘β-oxidation’ pathway (module M00087). This pathway encompasses a total of 13 genes, 11 of which were detected by RNAseq in most groups (except YG8-800 mice, with only ten genes detected). The majority of the genes were not differentially expressed; however, *Hadh* was downregulated in FXN^G127V^ female hearts and *Acaa1a* was upregulated in KIKO-700 female hearts (Fig. 5A). Furthermore, the levels of β-oxidation intermediates were mostly unchanged, except for a significant decrease in L-acetylcarnitine in FXN^G127V^ and KIKO-700 females (Fig. 5B). We also looked at the expression of genes of the KEGG ‘glycolysis’ pathway (module M00001). This pathway encompasses a total of 28 genes, 20 of which were detected by RNAseq in most groups (except YG8-800 mice, with only 18 genes detected). FXN^G127V^ females showed a significant increase in the expression of phosphofructokinase, platelet (*Pfkp*) (Fig. 5C). Notably, *Pfkp* overexpression was previously described in a model of HCM induced by pressure overload (Vigil-Garcia et al., 2021). In addition, we detected decreased expression of hexokinase 2 (*Hk2*) and enolase 3 (*Eno3*) in FXN^G127V^ females. In these mice, we found a significant reduction in glyceraldehyde 3-phosphate (G3P) levels (Fig. 5D), which were also decreased in FXN^G127V^ males and KIKO-700 females. FXN^G127V^ male hearts also showed a significant reduction in the level of pyruvate. KIKO-700 females had reduced levels of 3-phosphoglyceric acid (3PG) and phosphoenolpyruvic acid (PEP). Overall, these findings indicate that metabolic consequences of cardiac stress were manifested in several groups, but mostly in FXN^G127V^ female hearts.

### Transcriptional and metabolic profiles of ISR^mt^ are only evident in FXN^G127V^ hearts

**Fig. 6.**
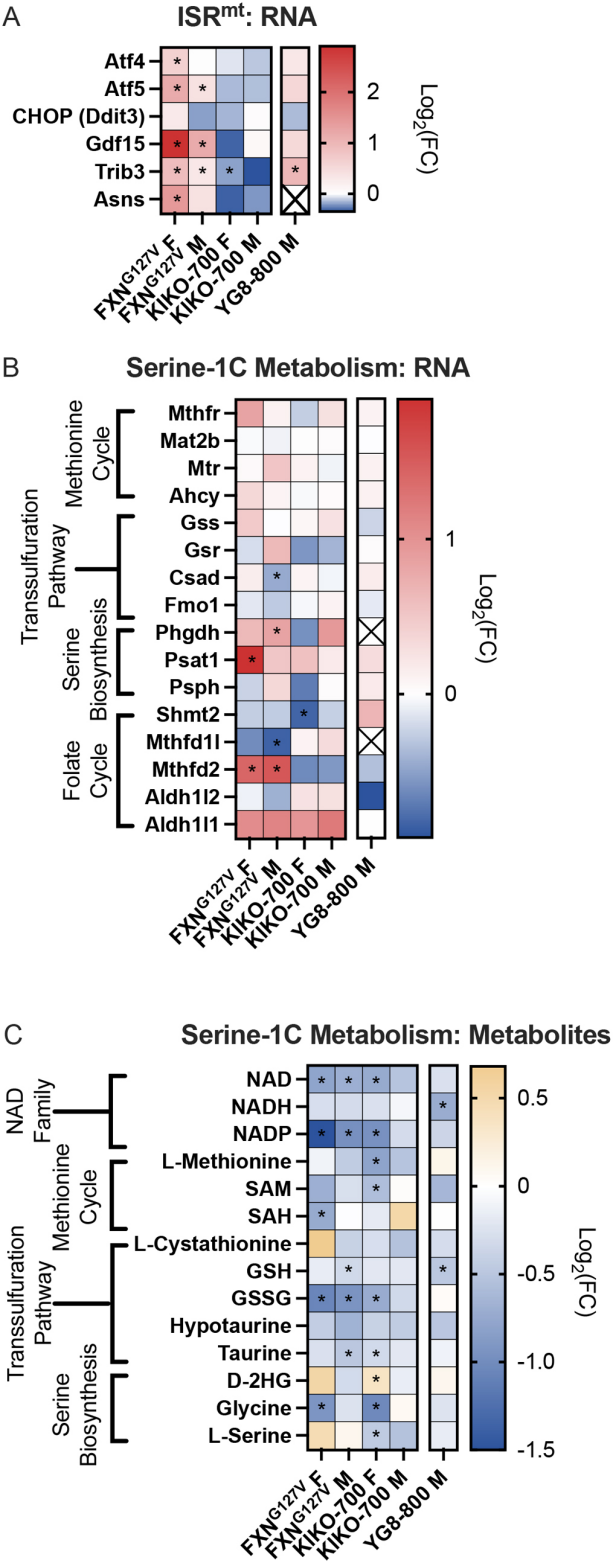
**Markers of ISR^mt^ activation and one-carbon (1C) metabolism alterations in FXN^G127V^ hearts.** (A,B) Heatmaps showing the expression of genes related to ISR^mt^ (A) and serine-1C metabolism (B). (C) Heatmap showing the abundance of metabolites related to serine-1C metabolism, including NAD family, methionine cycle, transsulfuration pathway and serine biosynthesis metabolites. *n*=3/sex/genotype 18-month-old FXN^G127V^ and KIKO-800 and *n*=4 18-month-old YG8-800 mice. Transcriptomics statistical analyses were performed with moderated *t*-test. Metabolomics statistical analyses were performed with unpaired two-tailed Student’s *t*-test, *P<0.05.

Unbiased integrated pathway analysis of the three models of FXN deficiency highlighted enrichment of pathways that may suggest ISR^mt^ activation in FXN^G127V^ and KIKO-700 hearts. Therefore, to characterize ISR^mt^-related pathways in depth, we first performed targeted analysis of the expression of canonical ISR^mt^-related genes (Forsstrom et al., 2019). In FXN^G127V^ hearts, there was upregulation of the expression of cytokine *Gdf15*, transcription factor *Trib3* and asparagine synthetase (*Asns*), indicative of cardiac ISR^mt^ activation (Fig. 6A) (Dogan et al., 2014; Forsstrom et al., 2019; Kaspar et al., 2021; Khan et al., 2017; Kuhl et al., 2017; Nikkanen et al., 2016; Sayles et al., 2022). We also found upregulation of the transcription factors *Atf4* in female FXN^G127V^ hearts and *Atf5* in male and female FXN^G127V^ hearts, but not in the other mouse models. Despite the observed transcriptional changes, we did not detect an increase in protein levels of ATF4 or ATF5 by western blotting in FXN^G127^ female and male hearts (Fig. S8A-C), probably due to translational regulation (Neill and Masson, 2023).

Metabolic remodeling associated with ISR^mt^ includes the upregulation of serine-1C metabolism (Mehrmohamadi et al., 2014; Nikkanen et al., 2016). We examined all genes of the serine-1C metabolism pathway and found that most of these genes were unchanged in the KIKO-700 and YG8-800 models (Fig. 6B). In FXN^G127V^ hearts, however, there was significant upregulation of serine biosynthesis pathway genes, *Phgdh* in males and *Psat1* in females, as well as the folate cycle gene *Mthfd2* in males and females (Fig. 6B). However, when we analyzed the expression of PSAT1 and MTHFD2 by western blotting, we only observed an increase in MTHFD2 in male FXN^G127V^ hearts (Fig. S8A,D,E). The elevation of serine-1C metabolism promotes the transsulfuration pathway for the methionine cycle to enhance glutathione (GSH) production and antioxidant defenses (Wu and Storey, 2021). However, the levels of GSH and oxidized glutathione (GSSG) in FXN^G127V^ hearts were decreased (Fig. 6C), suggesting that the glutathione biosynthetic pathway is unable to meet the demands of increased oxidative stress. Of note, NAD and NADP levels were decreased in all three models, by a greater degree in FXN^G127V^ hearts (Fig. 6C). Although the metabolic consequences of these alterations remain to be further elucidated, NAD depletion was reported in a mouse model of cardiac-specific FXN cKO in association with perturbations of SIRT1 activity and the NAD salvage pathway (Chiang et al., 2021). Next, we analyzed the expression of all detected GSH-linked antioxidant peroxidases (Gpx genes), S-transferases (Gst genes) and glutaredoxins (Glrx genes) and only found increased expression of *Gpx3* and *Gpx8* in FXN^G127V^ hearts (Fig. S9A). Owing to reduced glutathione and changes in glutathione metabolism gene expression in FXN^G127V^ hearts, we investigated protein glutathionylation, a posttranslational modification that protects proteins from irreversible oxidation (Andreadou et al., 2021). There was no statistically significant difference in protein glutathionylation between FXN^G127V^ and control hearts (Fig. S9B). Furthermore, we did not observe changes in malondialdehyde, a marker of lipid peroxidation (Fig. S9C). Next, we investigated the expression of other antioxidants, including peroxiredoxins (Prdx genes), superoxide dismutases (Sod genes) and NRF2-driven genes, and found no changes (Fig. S9D). In addition, we analyzed the expression of NADPH oxidase (NOX2/4) components, as increased expression of these enzymes has been associated with ISR^mt^ activation and linked to reactive oxygen species production and stress signaling (Chen et al., 2012; Nabeebaccus et al., 2017; Zhao et al., 2015), and did not see significant alterations in any of the models, except for *Ncf1* (NOX2 complex subunit) upregulation in FXN^G127V^ male hearts (Fig. S9E). Overall, we identified a transcriptional response associated with ISR^mt^ activation in FXN^G127V^ hearts. This moderate response seems to be capable of protecting the heart against oxidative stress, as shown by the absence of increased lipid peroxidation.

### Early ISR^mt^ activation in FXN^G127V^ hearts in the absence of defects in the activity of Fe-S cluster-dependent enzymes

**Fig. 7.**
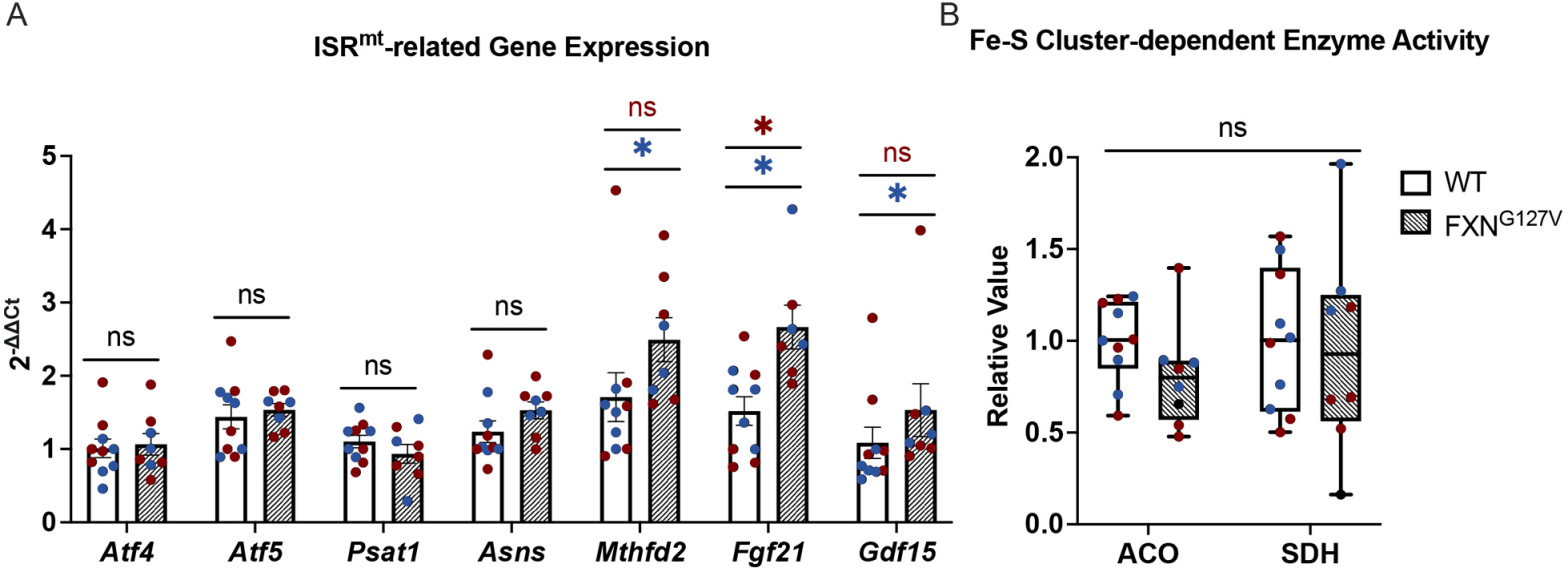
**Early markers of ISR^mt^ in male FXN^G127V^ hearts.** (A) mRNA expression of genes related to ISR^mt^ (*n*=5/sex 6-month-old mice, except *n*=3 for FXN^G127V^ males). Expression is normalized to *Gapdh* expression. Data are mean±s.e.m. (B) Activity of Fe-S cluster-dependent enzymes ACO and SDH in 6-month-old mice. Values are relative to those in WT littermate controls. Data are presented as a box-and-whisker plot: whiskers extend from the minimum to maximum values; lines indicate the median; boxes indicate the 25th and 75th percentiles. Blue circles represent male samples and maroon circles represent female samples. Statistical analyses were performed with unpaired two-tailed Student's *t*-test, *P<0.05; ns, not significant.

Previous studies have shown that ISR^mt^ progresses in temporal stages (Forsstrom et al., 2019) and, when chronically activated, leads to sustained metabolic alterations that contribute to cardiomyopathy (Sayles et al., 2022). Because 18-month-old FXN^G127V^ mice showed the most evidence of cardiac ISR^mt^ activation among the three mouse models we studied, we wanted to investigate ISR^mt^ markers at an earlier time point in these mice. At 6 months of age, we found an increase in the expression of *Fgf21* in female and male FXN^G127V^ hearts and upregulation of *Gdf15* and *Mthfd2* in male FXN^G127V^ hearts (Fig. 7A). However, we did not find alterations in the expression of other established ISR^mt^ genes, including *Atf4*, *Atf5*, *Psat1* and *Asns*. These findings are consistent with the initial disease phase of ISR^mt^, originally described in skeletal muscle (Forsstrom et al., 2019), in which elevation of a key mediator of metabolic remodeling, *Fgf21*, accompanied by increased *Gdf15* and *Mthfd2* expression, was shown to be involved in the progression of ISR^mt^.

In patients with FA, insufficient levels of FXN lead to decreased Fe-S cluster biogenesis, resulting in impairment of Fe-S cluster-dependent enzyme activity, including aconitase (ACO) and succinate dehydrogenase (SDH) (Puccio and Koenig, 2000; Rotig et al., 1997). Therefore, we wanted to investigate whether the severe FXN deficiency seen in FXN^G127V^ hearts (<1% residual FXN, Fig. 3A) causes an enzymatic defect that might contribute to mitochondrial dysfunction and ISR^mt^. Surprisingly, however, ACO and SDH activities were not decreased in FXN^G127V^ hearts compared to those in sex- and age-matched controls at 6 months (Fig. 7B). Together, these data suggest that very low levels of FXN are sufficient to maintain Fe-S-dependent enzymatic function for at least 6 months and that ISR^mt^ can be initiated independently of the function of these enzymes.

## DISCUSSION

The ISR^mt^ is an evolutionarily conserved adaptive response designed to help the heart face periods of acute stress (Eckl et al., 2021). However, if chronic and unresolved, as in the case of genetically determined mitochondrial diseases, it can become ‘maladaptive’ and cause detrimental metabolic imbalances that could contribute to heart failure (Smyrnias, 2021). One-carbon metabolism is a major metabolic pathway upregulated under ISR^mt^ conditions (Mehrmohamadi et al., 2014; Nikkanen et al., 2016). Interestingly, metabolic perturbations that included markers of dysregulated 1C metabolism were found in serum from FA patients (O'Connell et al., 2022). Although the cardiac origin of these markers remains to be established, these findings suggest the involvement of ISR^mt^ in FA. Furthermore, ISR^mt^ activation has been reported in models of genetic deletion or silencing of cardiac FXN (Huang et al., 2013; Tong et al., 2022; Vasquez-Trincado et al., 2021). Although these models suggest that chronic ISR^mt^ is associated with FXN loss, the complete loss of FXN does not accurately reflect the human disease, where there are variable amounts of residual FXN in the heart. Therefore, investigation of ISR^mt^ and a clearer understanding of its metabolic consequences in mouse models of FA that are genetically more representative of the condition in human patients is needed to determine whether this pathway could be targeted therapeutically. Here, we investigated cardiac ISR^mt^ in YG8-800, KIKO-700 and FXN^G127V^ mouse models, which have varying degrees of FXN deficiency. We chose to perform a multi-omic characterization of the heart of 18-month-old (aged) mice because these mice are viable up to this age and we wanted to maximize the probability of identifying biomarkers of mitochondrial stress.

The three models had varying degrees of cardiac FXN deficiency, with the most severe reduction observed in FXN^G127V^ hearts, in which there was ∼1% residual FXN. Interestingly, despite having longer GAA expansions, the levels of FXN in both YG8-800 and KIKO-700 hearts were comparable to the FXN levels reported in YG8sR (200 GAA repeats) (Anjomani Virmouni et al., 2015) and KIKO-230 (230 GAA repeats) (Miranda et al., 2002) mouse models. This suggests that mouse models of GAA expansion do not fully recapitulate the human disease, in which there is an inverse correlation between expansion size and levels of FXN (Koeppen, 2011).

Unbiased analyses of the transcriptomes and metabolomes of hearts from these FA mouse models identified OXPHOS and fatty acid metabolism among the most significantly altered pathways in YG8-800 hearts, while 1C and amino acid metabolism were among the most altered pathways in KIKO-700 and FXN^G127V^ hearts. Pyrimidine and glutathione biosynthesis were also altered in FXN^G127V^ hearts. Furthermore, we observed enrichment of pathways related to cardiac stress and cardiomyopathy in all three models. These findings provide evidence of moderate cardiac ISR^mt^-related perturbations in these mice, although severe cardiomyopathy symptoms have not been described in these mouse models. When we investigated individual markers of ISR^mt^, *Atf4/5* and *Mthfd2* were only upregulated in FXN^G127V^ hearts. The reason for differences in ISR^mt^ marker expression between FXN^G127V^ and the other mouse models remains to be fully elucidated. However, the most evident difference between the FXN^G127V^ and the YG8-800 and KIKO-700 mouse models is the level of residual FXN. It was suggested, based on findings in the inducible *Fxn* silencing model, that 20% residual FXN is sufficient to prevent OXPHOS impairment, cardiac dysfunction and related metabolic responses (Vasquez-Trincado et al., 2021). With the caveat that this model has postnatal silencing of *Fxn*, unlike the human disease, where patients presumably have reduced FXN levels since embryonic development, these data suggest that the threshold for cardiac involvement of ISR^mt^ is below 20% residual FXN in the mouse. Both the YG8-800 and KIKO-700 mouse models have more than 20% FXN in the heart, likely explaining the lack of alterations of cardiomyopathy and stress markers.

Hearts from the FXN^G127V^ mice displayed markers of ISR^mt^ progression. Based on a time-course analysis of ISR^mt^ in skeletal muscle of a mouse model of mitochondrial myopathy, early and late stages were described (Forsstrom et al., 2019). In the 6-month-old cohort of FXN^G127V^ male mice, we found increased expression of markers of early ISR^mt^, including *Mthfd2*, *Gdf15* and *Fgf21*. These markers were also shown to be upregulated prior to *Atf4/5* gene expression changes in a mouse model of mitochondrial cardiomyopathy (Sayles et al., 2022). Despite the extremely low levels of FXN in FXN^G127V^ hearts, the activities of Fe-S cluster-dependent enzymes ACO and SDH were unchanged at 6 months of age, suggesting that ISR^mt^ in the mouse heart can start prior to the onset of bioenergetic defects, like in a previously described model of mitochondrial cardiomyopathy (Sayles et al., 2022). At 18 months of age, both male and female FXN^G127V^ hearts express markers of ISR^mt^. The earlier activation of ISR^mt^ in males suggests sexual dimorphism due to unknown mechanisms. Future studies will investigate the time of onset of ISR^mt^ in the female FXN^G127V^ hearts and the potential role of sex hormones in delaying ISR^mt^ activation.

Our findings highlight important differences among the FXN^G127V^ model and the *Fxn* GAA repeat expansion models (YG8-800 and KIKO-700). We propose that only the FXN^G127V^ mutation causes sufficiently low levels of FXN in the mouse heart to trigger the upregulation of specific ISR^mt^ genes. The dramatic decrease (<1% residual FXN), however, did not result in a fatal cardiomyopathy, at least up to 18 months, unlike the cKO model, nor did it cause ACO and SDH enzymatic defects. Therefore, the mechanisms of ISR^mt^ induction in response to FXN loss in this model remain to be elucidated. A putative mechanism could involve defective FXN maturation due to the FXN^G127V^ mutation, which could be a source of proteotoxic stress in mitochondria. However, recent evidence indicates that residual G127V FXN retained its mitochondrial localization (Fil et al., 2023). Furthermore, studies of the human G130V FXN showed that this variant did not alter its interaction with Fe-S cluster biogenesis machinery (Schmucker et al., 2011). Therefore, even very low levels of FXN may be sufficient for mitochondrial Fe-S cluster biogenesis in the mouse heart.

In summary, we have shown that the ISR^mt^ may arise in the heart of mouse models with varying levels of FXN. However, there are limitations to these models, as they only develop a partial cardiac mitochondrial stress response, which complicate studies of mechanisms and clinical implications. Second, unlike in humans, the threshold for preservation of cardiac function appears to be as low as 1% residual FXN. Furthermore, the normal survival of these mice is very different from the disease outcome in humans. Lastly, unlike in FA patients, we did not observe an inverse correlation between GAA expansion and FXN levels in YG8-800 and KIKO-700 hearts. These findings further highlight the difficulty in modeling FA cardiomyopathy in murine models.

## MATERIALS AND METHODS

### Mouse models

All animal procedures were conducted in accordance with Weill Cornell Medicine, University of Alabama at Birmingham and Institut de Génétique et de Biologie Moléculaire et Cellulaire Animal Care and Use Committees, and were performed according to the Guidelines for the Care and Use of Laboratory Animals of the National Institutes of Health. YG8-800 mice were generated from the previously published YG8sR (Anjomani Virmouni et al., 2015) and are available from The Jackson Laboratory [*Fxn^em2.1Lutzy^* Tg(FXN)YG8Pook/800J, stock #030395]. CRISPR/Cas9-generated FXN^G127V^ mice were previously generated (Fil et al., 2020). Mice were euthanized by cervical dislocation.

The KIKO-700 mouse strain was generated using a standard homologous recombination approach on the C57BL/6 background. First, a 12.3 kbp genomic DNA used to construct the targeting vector was subcloned from a positively identified C57BL/6 fosmid clone (WI1-1938E18). The region was designed such that the 5′ homology arm extends ∼7.9 kbp to the human sequence with GAA repeats and the FRT-flanked neomycin (Neo) cassette. The 3′ homology arm extends ∼3.4 kbp from the Neo cassette. The human sequence with GAA repeats and Neo cassette was inserted 1,400 bp downstream of mouse *Fxn* exon 1. The human sequence containing ∼700 GAA repeats was amplified by PCR using genomic DNA isolated from FA patient cells as described (Li et al., 2016). The targeting vector was confirmed by restriction analysis and sequencing after each modification step. Subsequently, the construct containing ∼700 GAAs was transfected into FLP C57BL/6 embryonic stem cells (Ingenious) and positive clones were identified by neomycin selection. Genomic DNA was isolated from 400 embryonic stem cell clones and screened for correct integration and appropriate length of the GAA tract. Only 7% of clones harbored a tract of ∼700 GAAs. The selection cassette was removed using FLP recombinase. Five separate injection sets were conducted, resulting in successful generation of two chimeras. Subsequently, chimeras were bred with WT B6 mice to obtain fully heterozygous KI (*Fxn^+/700^*) animals. The FLP allele was removed by breeding with WT C57BL/6 animals. Finally, KI *Fxn^+/700^* mice were crossed with heterozygous *Fxn* mice with exon 4 deleted [*Fxn^+/−^*; B6.129(Cg)-*Fxn^tm1Mkn^*/J, stock #016842, The Jackson Laboratory] to obtain *Fxn^700/−^* (KIKO-700) study animals. All animals included in this study were genotyped, and the length of the GAA repeats was verified by repeat PCR. No significant germline instability was observed.

### RNAseq

RNA was extracted from heart tissue using TRIzol (Life Technology) and an RNeasy Mini Kit (Qiagen), according to the manufacturers’ instructions. 3′ RNAseq libraries were prepared from 500 ng RNA per sample using a Lexogen QuantSeq 3′ mRNA-Seq Library Prep Kit FWD for Illumina and pooled for reduced run variability. Libraries were sequenced with single-end 86 bp on an Illumina NextSeq500 sequencer (Cornell Genomics Facility). All computations were performed in the R statistical environment (version >4.2.0; https://www.R-project.org/). Raw sequence reads were processed using ShortRead package (version 1.54.0) (Morgan et al., 2009). Trimmed reads were aligned to the mouse genome assembly GRCm38.94 using the Rsubread package (version 2.10.5) (Liao et al., 2019) with default parameter settings. The Rsubread::featureCounts function was used to assign mapped stranded sequencing reads to genes and count features. The Limma package (version 3.52.1) (Ritchie et al., 2015) was used to obtain normalized and variance-stabilized counts and to calculate differential gene expression. Pathway analysis for all gene expression data was performed with the gprofiler2 (Kolberg et al., 2020) and clusterProfiler (Wu et al., 2021) packages, using the GO Biological Process and KEGG databases. A false discovery rate-corrected *P*-value of <0.05 was used to determine significance. Pathways shown in the figures were condensed using the simplify function from the clusterProfiler package (Wu et al., 2021) to merge terms with more than 40% overlapping annotated genes.

### Metabolomics

Untargeted metabolomics of heart tissue was performed at Weill Cornell Medicine Meyer Cancer Center Proteomics and Metabolomics Core Facility. Briefly, 15 mg of cardiac tissue was homogenized in 80% methanol (Sigma-Aldrich) using Tissue Tearer (BioSpec) on dry ice. Samples were incubated at −80°C for 4 h. Homogenates were then centrifuged at 14,000 ***g*** for 20 min at 4°C. The supernatant was extracted and injected into liquid chromatography-mass spectrometry apparatus to measure mass-to-charge ratio. The mass spectrometry data were processed using Compound Discoverer (Thermo Fisher Scientific). An in-house Human Metabolome Database metabolite library was searched for metabolite identification based on accurate mass. Analysis of metabolite changes was performed with MetaboAnalyst (version 5.0) (Xia et al., 2009), which included the following: fold change analyses, heatmap generation, pathway enrichment analysis and joint-pathway impact analysis. Joint-pathway impact analysis was performed with hypergeometric test and with degree centrality topology applied.

### ELISA

Human or mouse FXN levels were measured by ELISA (for human, Abcam, ab176112; for mouse, Abcam, ab199078) according to the manufacturer's instructions. Briefly, protein was extracted from homogenized heart tissue using 1X Cell Extraction Buffer PTR from the relevant ELISA kit. Standards or protein lysates were incubated with the antibody cocktail for 1 h at room temperature on a plate shaker set to 400 rpm. Following washing and addition of TMB Development Solution and Stop Solution from the relevant ELISA kit, absorbance was recorded at 450 nm in a PowerWave XS (Biotek). Human and mouse FXN levels were normalized to their respective standard curve measurements.

### Western blotting

Hearts were digested in RIPA buffer (20 mM Tris-HCl pH 7.5, 150 mM NaCl, 1 mM Na_2_EDTA, 1 mM EGTA, 1% NP-40, 1% sodium deoxycholate, 2.5 mM sodium pyrophosphate, 1 mM beta-glycerophosphate, 1 mM Na_3_VO_4_, 1 µg/ml leupeptin; Cell Signaling Technology), and protein concentration was determined by the Bradford protein assay (Bio-Rad). Total heart lysates (25, 75 or 100 μg) were denatured in 1× Laemmli buffer (Bio-Rad) containing 2-mercaptoethanol (Sigma-Aldrich) at 95°C for 10 min, separated by electrophoresis in a 4-12% SDS-PAGE gel (Bio-Rad) and transferred to a PVDF membrane (Bio-Rad). For protein glutathionylation detection, total heart lysates (5 μg) were denatured but not reduced in 1× Laemmli buffer (without 2-mercaptoethanol), separated by electrophoresis in a 4-12% NuPAGE gel (Thermo Fisher Scientific) using MOPS buffer (Thermo Fisher Scientific) and transferred to a nitrocellulose membrane (Bio-Rad). Blots were incubated in 3% bovine serum albumin (BSA) in Tris-buffered saline (20 mM Tris, 150 mM NaCl, pH 7.6) with 1% Tween 20 for 1 h at room temperature. Primary antibodies were incubated overnight at 4°C. Secondary antibodies were incubated for 45 min at room temperature. For all blots, proteins were detected using Clarity Western ECL Blotting Substrates (Bio-Rad) and imaged on a ChemiDoc Touch (Bio-Rad). Normalization of FXN levels was determined by Ponceau S staining (Sigma-Aldrich); the remaining western blots were normalized to either GAPDH (mouse monoclonal; 1:1000; ProteinTech, 60004) or TIM23 (mouse monoclonal; 1:1000; BD Transduction Laboratories, 611222) expression. The following antibodies were used: monoclonal mouse anti-FXN (1:1000; Millipore Sigma, clone 4F9, MABN2313), polyclonal rabbit anti-FXN (1:1000; ProteinTech, 14147-1-AP), monoclonal mouse anti-ATF4 (1:1000; ProteinTech, 60035), polyclonal rabbit-anti-ATF5 (1:1000; Abcam, ab184923), polyclonal rabbit anti-PSAT1 (1:1000; ProteinTech, 10501-1-AP), polyclonal rabbit anti-MTHFD2 (1:1000; ProteinTech, 12270-AP) and monoclonal mouse anti-GSH (1:1000; ViroGen, 101-A).

### Lipid peroxidation assay

Lipid peroxidation was measured using a Lipid Peroxidation (MDA) Assay Kit (Abcam, ab118970) according to the manufacturer's instructions. Briefly, 60 μg total heart lysate was incubated with TBA reagent (from the MDA Assay Kit) at 95°C for 60 min, then brought to room temperature, generating MDA-TBA adduct. Samples were then added to a 96-well microplate, and absorbance was measured using a microplate reader (Molecular Devices, SpectraMAX) at 532 nm wavelength. Absorbance was normalized to a standard curve of known MDA concentrations.

### Quantitative PCR

RNA was extracted from heart tissue using TriZol (Life Technology), according to the manufacturer's instructions. Total mRNA (10 µg) was used for reverse transcription with SuperScript IV Reverse Transcriptase (Thermo Fisher Scientific) in 100 µM DTT, 50 µM oligo dT, 10 mM dNTP and 40 U/µl RNAsin in a total volume of 10 µl. A PCR was performed to amplify exons. Primer sequences can be found in Table S4. Quantification of the reverse transcription PCR product was obtained on a LightCycler 480 (Roche).

### Enzymatic activity assays

ACO and SDH activity were measured as previously described (Puccio et al., 2001). Briefly, proteins were isolated from 10 mg heart tissue in 50 µl extraction buffer (10 mM KH_2_PO_4_, 2 mM EDTA and 1 mg/ml BSA). ACO activity was measured at 240 nm following the addition of 5 mM citrate in Buffer S (150 mM Tris-HCl, pH 7.4). SDH activity was measured at 600 nm following the addition of 200 mM ATP, 320 mM potassium cyanide (KCN), 5 mM succinate, 0.05 mM decylubiquinone in Buffer S. SDH activity was normalized to isocitrate dehydrogenase activity. All reagents were from Sigma-Aldrich, except for KCN (Prolabo). Activity was measured on a Cary 50 Scan UV Visible Spectrophotometer (Varian).

### Data analysis

The number of animals (biological replicates) for transcriptomics and metabolomics performed at 18 months was *n*=3/sex/genotype for FXN^G127V^ and KIKO-700 and *n*=4/genotype for YG8-800. Biological replicates for enzymatic assays and quantitative PCR were *n*=5/sex/genotype (except FXN^G127V^ males, which were *n*=3) at 6 months. Biological replicates for western blots and lipid peroxidation were *n*=2/sex/genotype. Statistical analyses were performed using Prism (GraphPad Software, version 9.1.1). Two-group comparisons were analyzed by unpaired two-tailed Student's *t-*test. Differences were considered statistically significant with a *P*-value <0.05. Data are presented as the mean±s.e.m. Pathway analysis was performed using MetaboAnalyst using the hypergeometric test and relative-betweenness centrality. Joint-pathway analysis was performed with hypergeometric test and degree centrality topology applied, and these data were integrated based on pathway level combined *P*-values for all pathways.

## Supplementary Material

10.1242/dmm.050114_sup1 Supplementary information
