# Correction: Comparative multi-omic analyses of cardiac mitochondrial stress in three mouse models of frataxin deficiency

**DOI:** 10.1242/dmm.050689

**Published:** 2024-01-23

**Authors:** Nicole M. Sayles, Jill S. Napierala, Josef Anrather, Nadège Diedhiou, Jixue Li, Marek Napierala, Hélène Puccio, Giovanni Manfredi

There was an error published in *Dis. Model. Mech.* **16**, dmm050114 (doi:10.1242/dmm.050114).

**Fig. 2C (corrected panel).**
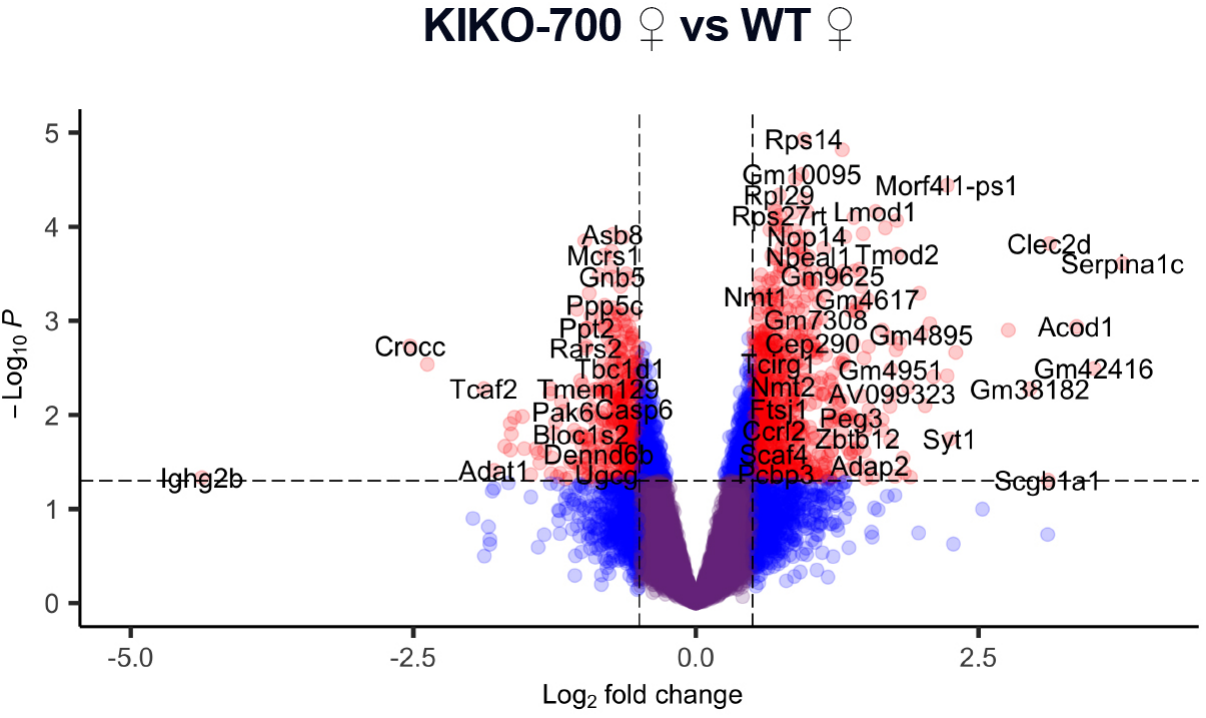
**Enrichment of pathways associated with mitochondrial integrated stress response (ISR^mt^) and cardiac stress in KIKO-700 mice.** (C,D) Volcano plot of gene expression in KIKO-700 females (C) and males (D) (*n*=3/sex/genotype 18-month-old mice). Genes in red indicate *P*>0.05, |FC|>0.5.

**Fig. 2C (original panel).**
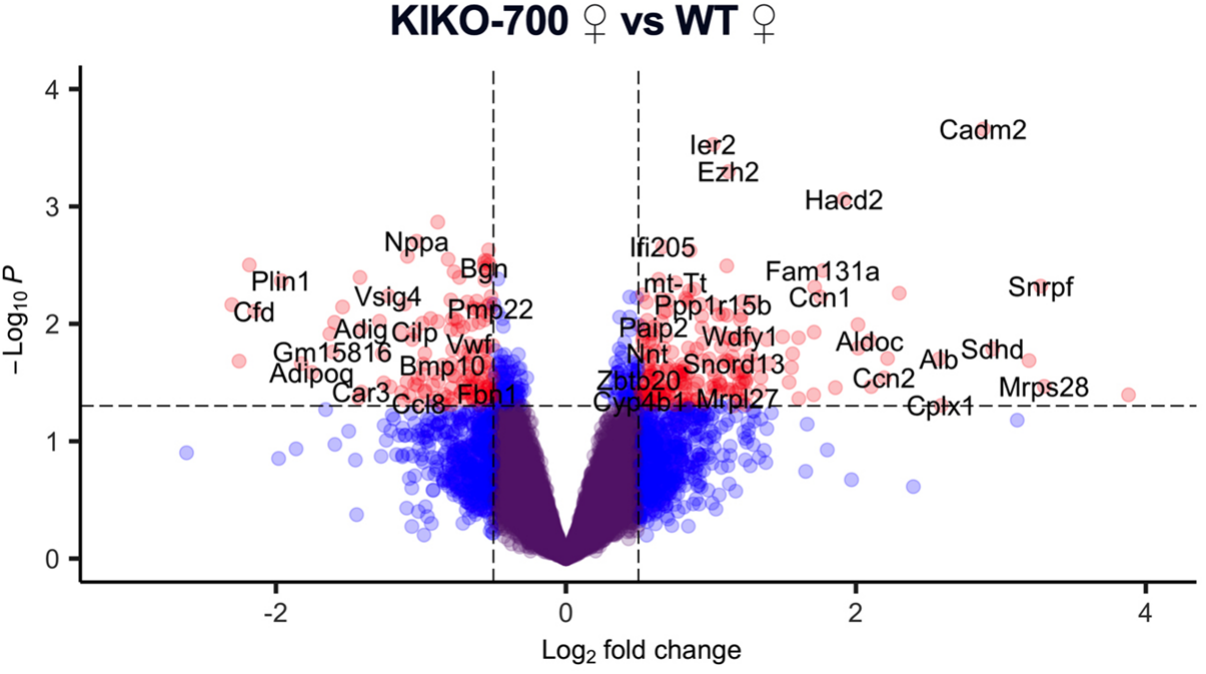
**Enrichment of pathways associated with mitochondrial integrated stress response (ISR^mt^) and cardiac stress in KIKO-700 mice.** (C,D) Volcano plot of gene expression in KIKO-700 females (C) and males (D) (*n*=3/sex/genotype 18-month-old mice). Genes in red indicate *P*>0.05, |FC|>0.5.

In Fig. 2, panel C was a replicate of panel D. Fig. 2C has been amended to include the correct volcano plot illustrating gene expression in female KIKO-700 hearts.

The corrected and original figure panels are shown below. Both the online full-text and PDF versions of the article have been updated.

The authors apologise for the error and any inconvenience it may have caused.

