# Supplementary material for: Comparative multi-omic analyses of cardiac mitochondrial stress in three mouse models of frataxin deficiency
Source: Dis Model Mech. 2023 Oct 9;16(10):dmm050114. doi: 10.1242/dmm.050114 (PMC10581388; doi:10.1242/dmm.050114)
Supplement: Supplementary information [file dmm-16-050114-s1.pdf]

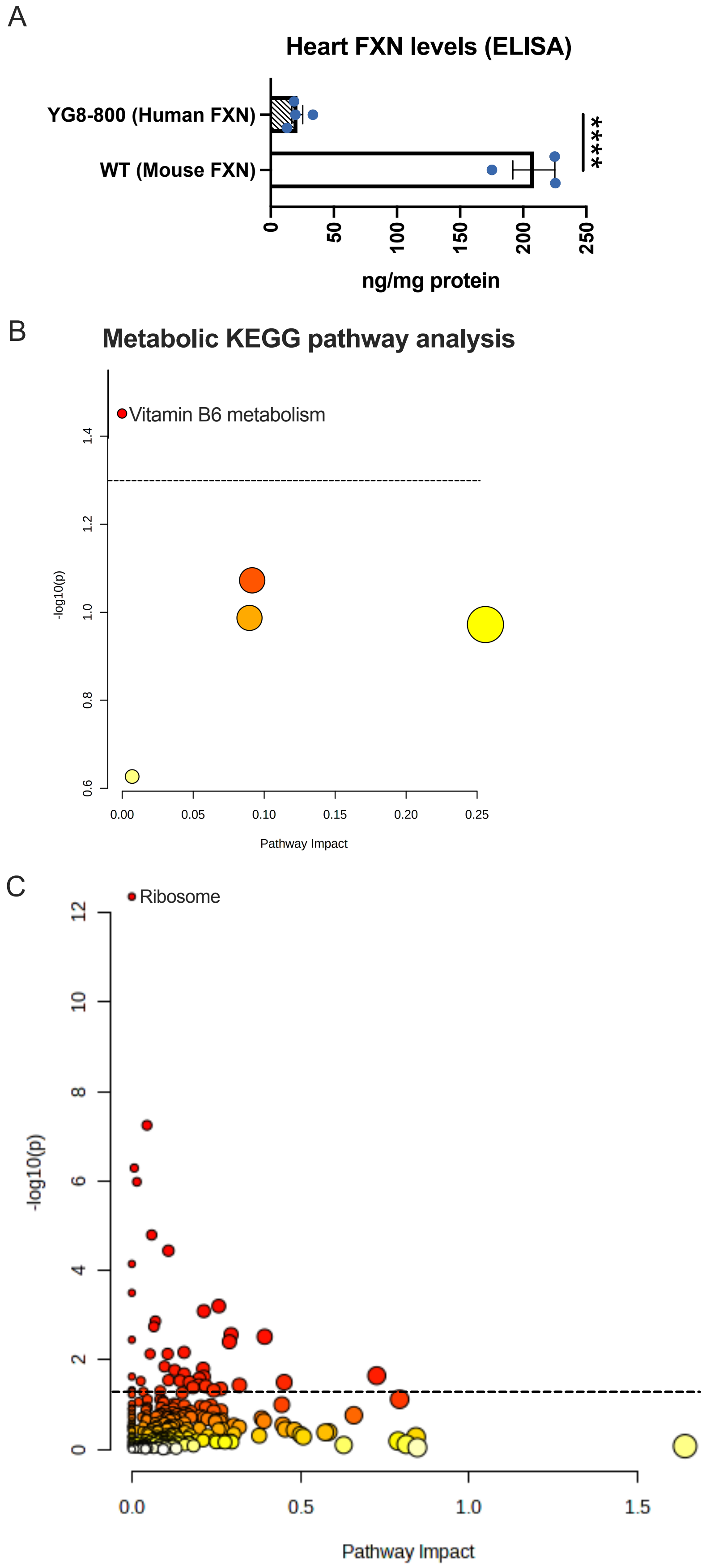

Fig. S1. Ribosomal pathway enrichment in YG8-800 joint-pathway analysis. (A) Measurement of mouse and human FXN by ELISA (statistical analysis was performed with unpaired two-tailed Student's t-test, ****P<0.0001, data represented as mean ± SEM). (B) Unbiased metabolic KEGG pathway analysis. Significantly enriched pathways (-Log10(p) > 1.3) are labeled. (C) Integrated metabolomics and transcriptomics data from YG8-800 males including ribosomal genes. n=4/ genotype 18-months old mice.

Disease Models & Mechanisms • Supplementary information

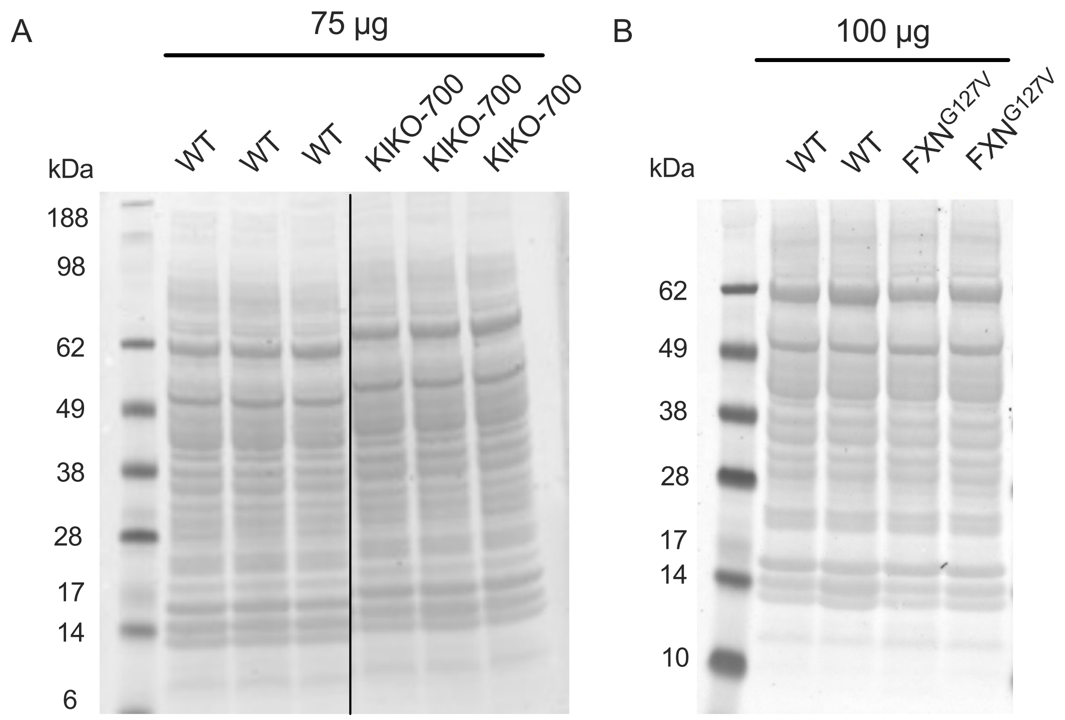

Fig. S2. FXN normalization blots. (A) Ponceau S staining of the membrane for KIKO-700 samples in Fig. 2A (*n*=3) (B) Ponceau S staining of the membrane for FXN^{G127V} samples, where 100 µg were loaded in Fig. 3A (*n*=2).

Disease Models & Mechanisms • Supplementary information

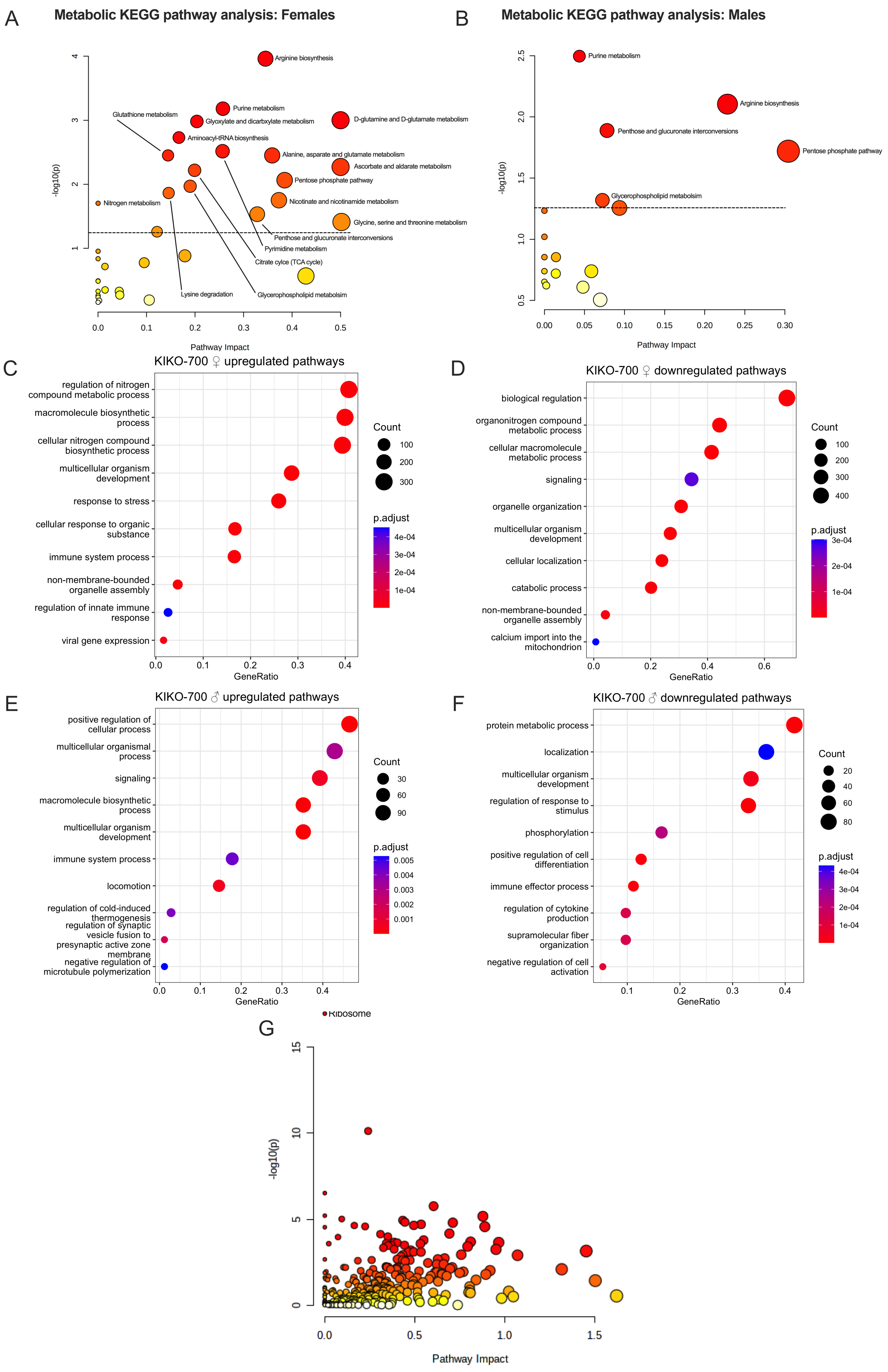

Fig. S3. Enrichment of transcriptional and metabolic pathways associated with metabolic stress in KIKO-700 hearts. (A, B) Unbiased metabolic KEGG pathway analysis for females (A) and males (B). (C, D) Top 10 results of unbiased GO analyses of significantly upregulated (C) or downregulated (D) genes in female KIKO-700 hearts. (E, F) Top 10 results of unbiased GO analyses of significantly upregulated (E) or downregulated (F) genes in male KIKO-700 hearts. (G) Integrated metabolomics and transcriptomics data from KIKO-700 females including ribosomal genes. *n*=3/sex/genotype 18-months old mice.

Disease Models & Mechanisms • Supplementary information

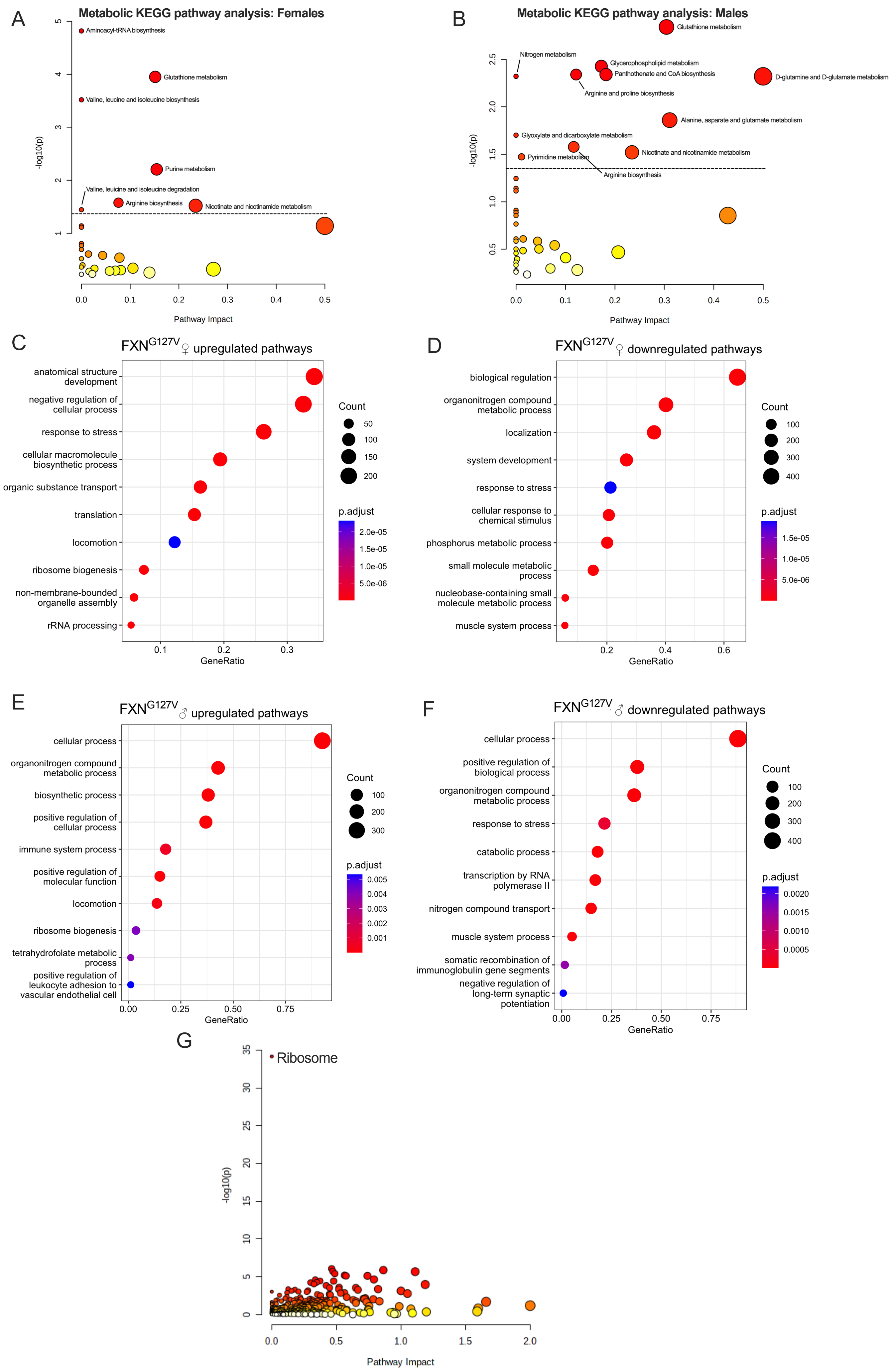

Fig. S4. Enrichment of transcriptional and metabolic pathways associated with cardiac stress and ISR^{mt} in FXN^{G127V} hearts. (A, B) Unbiased metabolic KEGG pathway analysis for females (A) and males (B). (C, D) Top 10 results of unbiased GO analyses of significantly upregulated (C) or downregulated (D) genes in female FXN^{G127V} hearts. (E, F) Top 10 results of unbiased GO analyses of significantly upregulated (E) or downregulated (F) genes in male FXN^{G127V} hearts. (G) Integrated metabolomics and transcriptomics data from FXN^{G127V} females including ribosomal genes. *n*=3/sex/genotype 18-months old mice.

Disease Models & Mechanisms • Supplementary information

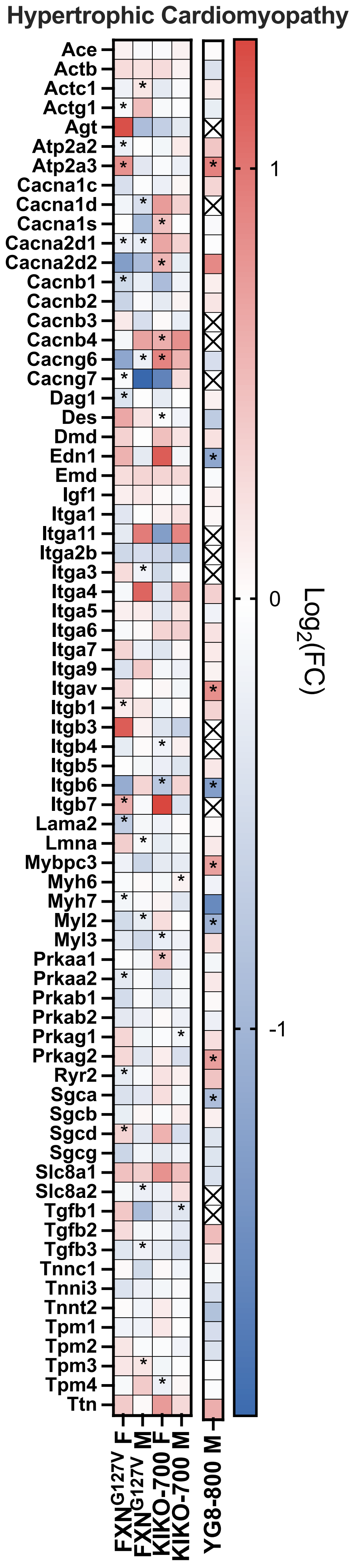

Fig. S5. Lack of overall expression changes in hypertrophic cardiomyopathy in all three FXN-deficiency models. Targeted gene expression analysis of the KEGG pathway “Hypertrophic Cardiomyopathy” (mmu05410) represented in a heatmap, in which red indicates increased gene expression and blue indicates decreased gene expression. ‘X’ indicates that a gene was not detected by RNAseq. F, female; M, male. Expression is represented as Log2[fold change (FC)]. *n*=3/sex/genotype 18-months old FXN^{G127V} and KIKO-800 and n=4 18-months old YG8-800 mice. Transcriptomics statistical analyses were performed with moderated t-test. *P<0.05.

Disease Models & Mechanisms • Supplementary information

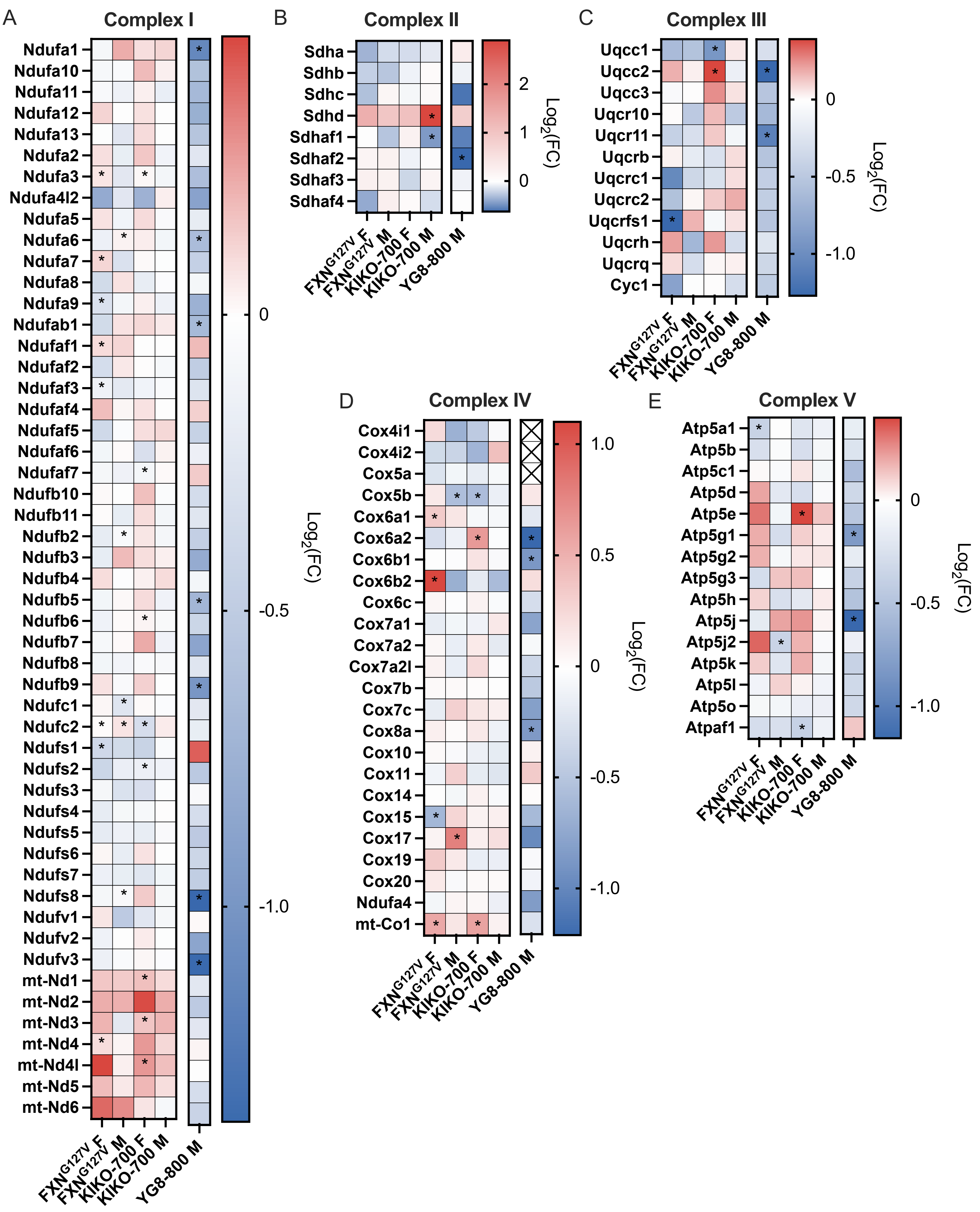

Fig. S6. OXPHOS subunit expression is largely unchanged in the three FXN-deficiency models. (A-E) Heatmaps showing the expression of genes related to Complex I (A), Complex II (B), Complex III (C), Complex IV (D), and Complex V (E). *n*=3/sex/genotype 18-months old FXN^{G127V} and KIKO-800 and *n*=4 18-months old YG8-800 mice. Transcriptomics statistical analyses were performed with moderated t-test. **P*<0.05.

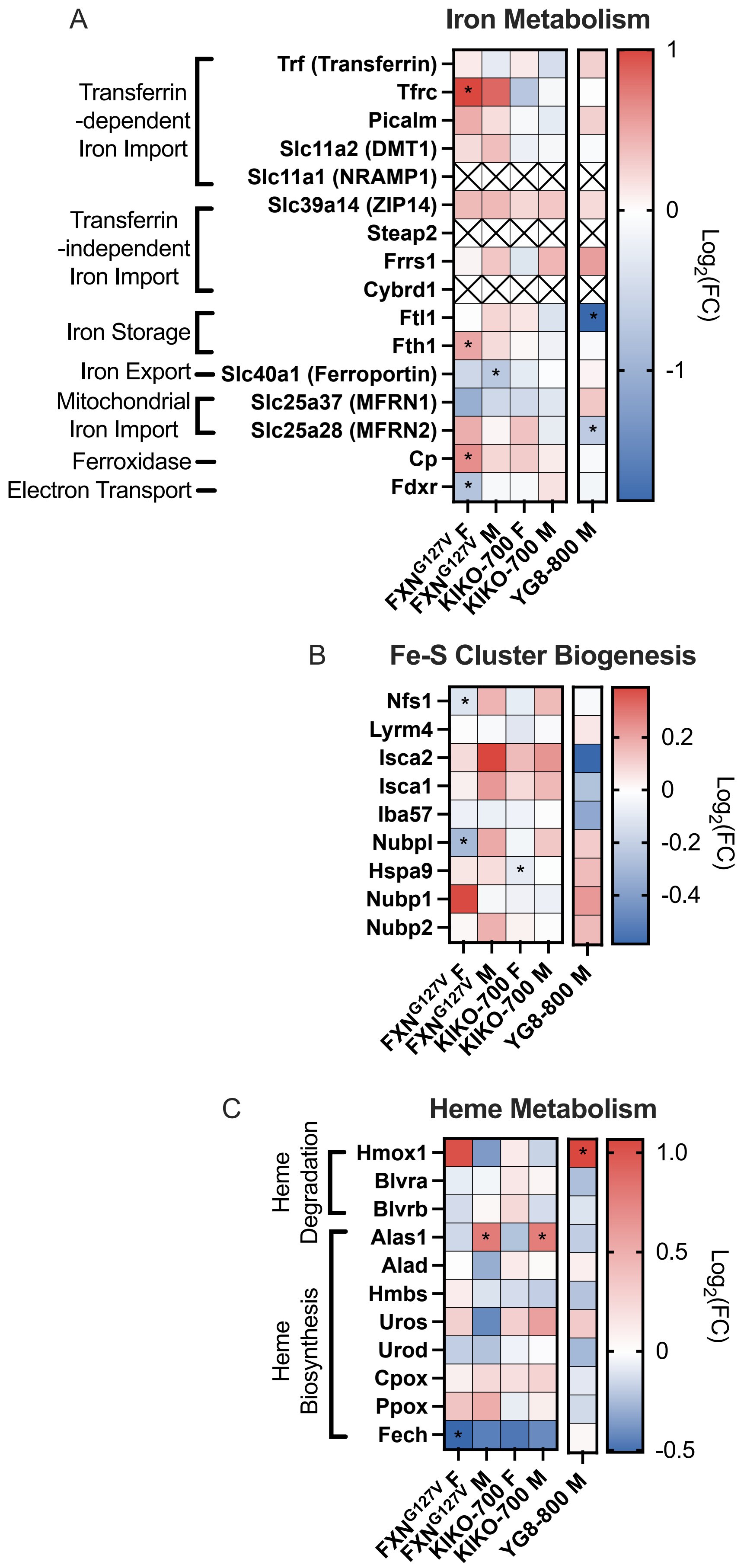

Fig. S7. Iron metabolism gene expression is unchanged in the three FXN-deficiency models. (A-C) Heatmap showing the expression of genes related to iron metabolism (A), Fe-S cluster biogenesis (B), and heme metabolism (C). *n*=3/sex/genotype 18-months old FXN^{G127V} and KIKO-800 and *n*=4 18-months old YG8-800 mice. Transcriptomics statistical analyses were performed with moderated t-test. **P*<0.05.

Disease Models & Mechanisms • Supplementary information

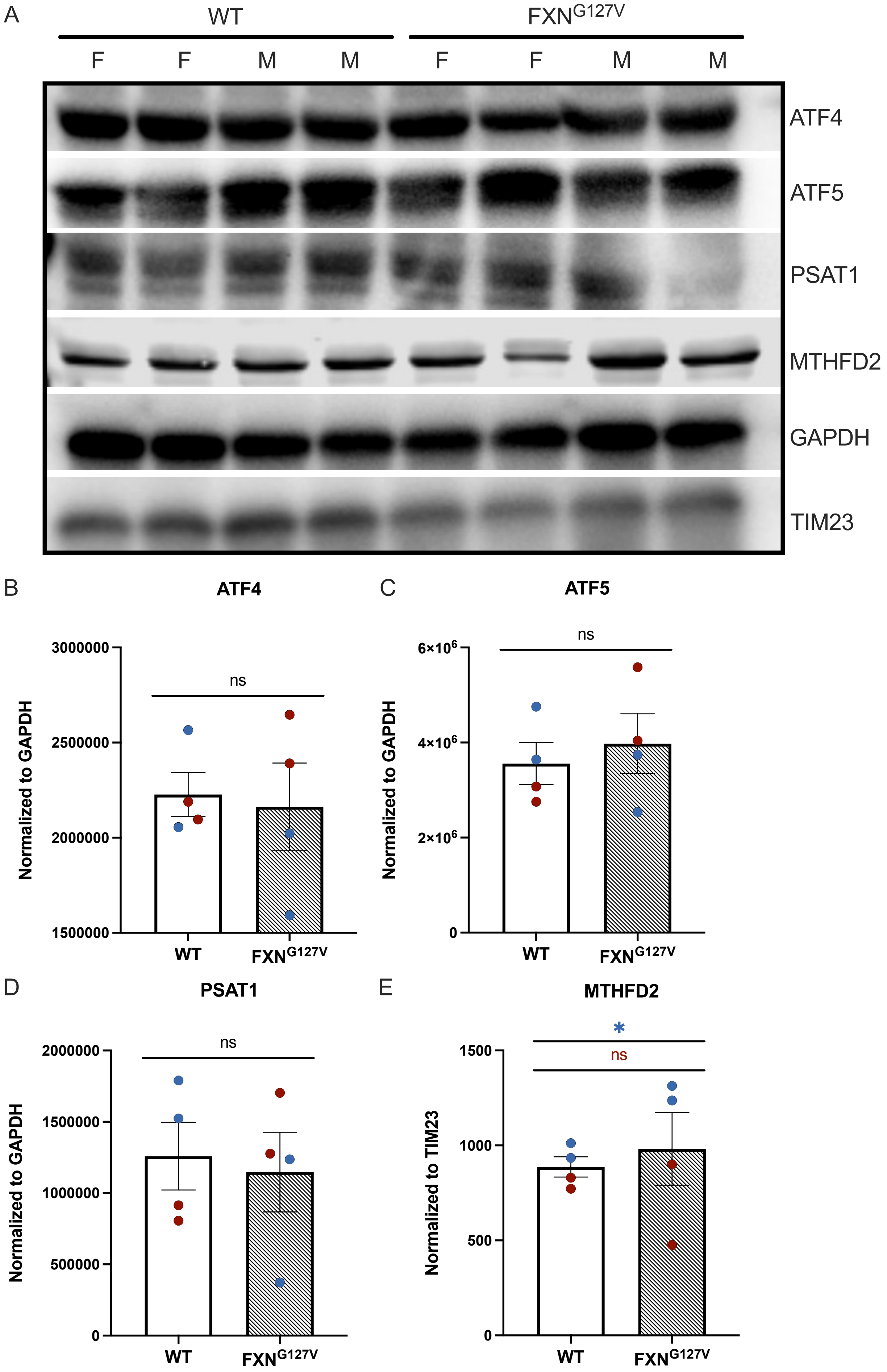

Fig. S8. Levels of ISR^{mt} marker proteins in FXN^{G127V} hearts. (A) Western blot analyses of female (F) and male (M) FXN^{G127V} heart lysates for ATF4, ATF5, PSAT1, and MTHFD2 and normalization protein GAPDH (or TIM23 for MTHFD2). (B-E) Quantification of Western blots for ATF4 (B), ATF5 (C), PSAT1 (D), and MTHFD2 (E). Maroon circles denote female samples and blue circles denote male samples. Statistical analysis was performed with unpaired two-tailed Student's *t*-test, data represented as mean ± SEM, *n*=4/genotype (*n*=2/sex) 18-months old mice. *P<0.05.

Disease Models & Mechanisms • Supplementary information

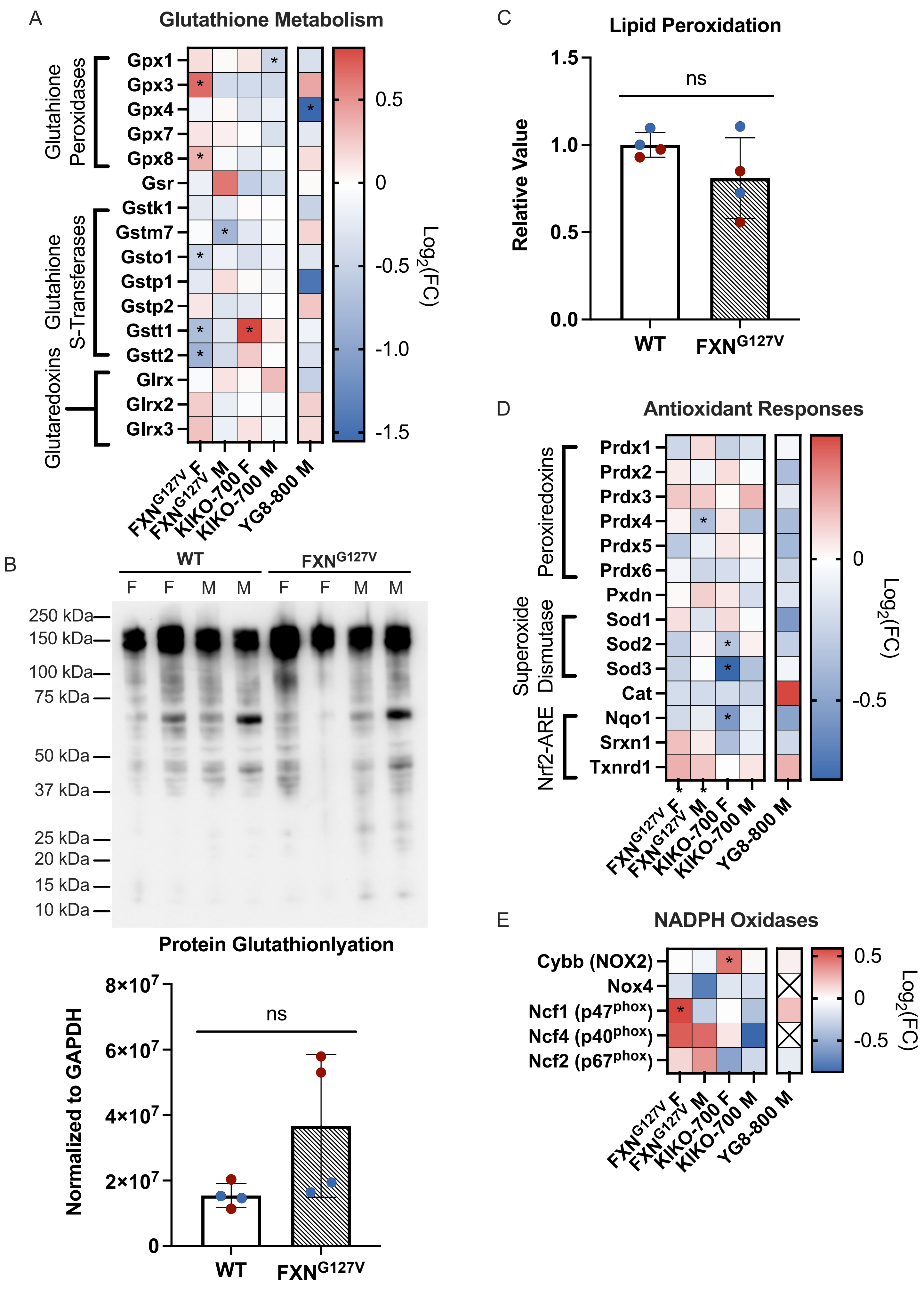

Disease Models & Mechanisms • Supplementary information

Fig. S9. Absence of oxidative stress markers in all three FXN-deficiency models. (A) Heatmap showing the expression of genes related to glutathione metabolism. (B) Western blot of protein glutathionylation in WT FXN^{G127V} heart lysates (5 µg), quantified below. Red circles denote female samples and blue circles denote male samples. (C) Relative absorbance values at 532nm in WT and FXN^{G127V} heart lysates. (D, E) Heatmap showing the expression of genes related to antioxidant responses (D) and NADPH oxidases (E). For heatmaps, *n*=3/sex/genotype 18-months old FXN^{G127V} and KIKO-800 and *n*=4 18-months old YG8-800 mice. For protein glutathionylation and lipid peroxidation, *n*=4/genotype (*n*=2/sex) 18-months old mice. Transcriptomics statistical analyses were performed with moderated *t*-test. The remaining statistical analyses were performed with unpaired two-tailed Student's *t*-test, data represented as mean ± SEM. *P<0.05.

Table S1. Unbiased metabolic KEGG pathway anlayses of significantly altered metabolites in YG8-800, KIKO-700, and FXNG127V hearts.

Click here to download Table S1

Table S2. Unbiased metabolic joint-pathway analyses of DEGs and significantly altered metabolites in YG8-800, KIKO-700, and FXNG127V hearts.

Click here to download Table S2

Table S3. Comparisons of enriched joint-pathway results between YG8-800, KIKO-700, and FXNG127V hearts.

Click here to download Table S3

Disease Models & Mechanisms • Supplementary information

Table S4. Quantitative PCR primer sequences

Gene	Forward primer	Reverse primer
Fgf21	ACCTCTACACAGATGACGACCA	AGAAACCTAGAGGCTTTGACACC
Gdf15	GCTGCTACTCCGCGTCAACC	CTACCCGTAAGCGCAGTTCC
Psat1	ACTACAAAGTGCAGGCTGGG	TTGATCCATTCCAGGACCAT
Mthfd2	AATTTGGGCTTTGCAGTGAC	ACACTCCCAAAGAGCAGCTG
Asns	ATTACGACAGTTCGGGCATC	TCTCAGTTCGAGACCGTGTC
Atf4	GGCCAAGCACTTGAAACCTC	TCCTCCTTGCCGGTGTCTGA
Atf5	TCCTCCACCTTCGCCTT	GAGTTTCCCATAGTCTACGAGCC
Gapdh	TTGTGATGGGTGTGAACCAC	TTCAGCTCTGGGATGACCTT

Disease Models & Mechanisms • Supplementary information
